# Pharmacokinetic Profile and Oral Bioavailability of Diosgenin, Charantin, and Hydroxychalcone From a Polyherbal Formulation

**DOI:** 10.3389/fphar.2021.629272

**Published:** 2021-04-29

**Authors:** Ruchira Salunkhe, Chhaya Gadgoli, Archana Naik, Nikita Patil

**Affiliations:** Saraswathi Vidya Bhavan’s College of Pharmacy, Dombivli, India

**Keywords:** diosgenin, charantin, hydroxychalcone, bioavailability, pharmacokinetics, antidiabetic, herbal formulations

## Abstract

**Background:** Diosgenin, charantin, and hydroxychalcone are utilized for standardization of popular antidiabetic herbal drugs *Trigonella foenum-graecum* L*.* belonging to family Fabaceae, *Momordica charantia* L*.* belonging to family Cucurbitaceae, and *Cinnamomum verum* J. Presl belonging to family Lauraceae. However, no reports on the bioavailability of these markers were available. The present study was undertaken to determine the bioavailability and pharmacokinetic profile of the markers and formulations containing the herbs.

**Methods:** The pharmacokinetic profile and absolute bioavailability of the pure active markers were determined in male Wistar rats by administrating individually the doses of 1.5 mg/kg i.v. and 15 mg/kg p.o., followed by estimation of serum levels of the markers at 0, 10, 30, 60, 120, and 240 mins till 24 h time points by a validated bioanalytical HPTLC method. Two standardized antidiabetic capsule formulations containing spray dried hydroalcoholic extracts of seeds of *Trigonella foenum-graecum* L. (42.8 mg equivalent to 0.95%w/w of diosgenin), fresh fruits of *Momordica charantia* L. (21.4 mg equivalent to 0.4% w/w of charantin), and bark of *Cinnamomum verum* J. Presl (10.71 mg equivalent to 0.079 %w/w hydroxychalcone) were prepared. In one formulation, piperine 1.5 mg was added along with the other herbal extracts mentioned. Bioavailability and pharmacokinetic profile of these two formulations were determined in male Wistar rats through estimating serum levels of active markers diosgenin, charantin, and hydroxychalcone at 0, 10, 30, 60, 120, and 240 mins till 24 h later oral administration of the formulations (Formulation without piperine F1 and formulation with Piperine F2).

**Results:** Plasma concentrations were found to decline mono-exponentially following intravenous administration, and the mean elimination half-life (t_1/2_) was observed to be 7.93, 8.21, and 4.66 h, respectively. The absolute oral bioavailability of pure markers was observed to be 9.0 ± 0.2%, 8.18 ± 0.36%, and 10.54 ± 0.52% by the dose normalization method. The oral bioavailabilities of the formulations with respect to diosgenin, charantin, and hydroxychalcone were found to be 9.78, 10.743, and 8.07%, respectively. The formulation containing piperine indicated a significant (*p* < 0.01) increase in the bioavailabilities of all the marker compounds.

**Conclusion:** In conclusion, diosgenin and charantin have low bioavailabilities as compared to hydroxychalcone. The bioavailabilities of all the three marker compounds can be increased exponentially with the addition of piperine.

## Introduction

The seeds of *Trigonella foenum-graecum* L, fruits of *Momordica charantia* L, and the bark of *Cinnamomum verum* J. Presl have been reported to possess antidiabetic activity (Rajesh et al., 2016; Mahmoud et al., 2017; Geberemeskel et al., 2019). Around 75% of herbal formulations available in Indian market for treatment of diabetes contain these herbs. Diosgenin from seeds of *T. foenum-graecum* is reported to have antidiabetic activity in the long-term use by restoration of pancreatic *β*-cells, downregulation of enzymes involved in hepatic gluconeogenesis and glucose export, upregulation of hepatic glucokinase, and increase in the amounts of hepatoprotective and antioxidant enzymes (Jesus et al., 2016). Charantin from fruits of *M. charantia* acts through stimulating peripheral and skeletal muscle glucose utilization, inhibition of adipocyte differentiation, suppression of key gluconeogenic enzymes, stimulation of key enzyme of the HMP pathway, and preservation of islet *β* cells and their functions (Nagappan et al., 2018). Hydroxychalcone from the bark of *C. verum* acts by mimicking the effect of insulin through enhancing glucose uptake and phosphorylation of insulin receptor in adipocytes; moreover, hydroxychalcone was also reported as a potential dietary PPARγ ligand (Eissa et al., 2017).

The pharmacological actions of any drug are dependent on the serum levels of the same. The appropriate serum levels of a drug are dependent on its absorption and distribution metabolism of the drug. The bioavailability of a drug is defined as measurement of the rate and extent to which a drug reaches at the site of action (Kumar and Sanjita, 2013). In case of extracts of herbs or herbal drugs, it is challenging to determine bioavailability due to presence of many constituents. There is very little or no data available revealing pharmacokinetics and bioavailability of phytoconstituents and the formulations containing herbal extracts thereof. Such studies not only help in understanding the efficacy of formulations but also helpful in improving efficacy of formulation through adapting various ways of increasing bioavailability of phytoconstituents.

**FIGURE 1:**
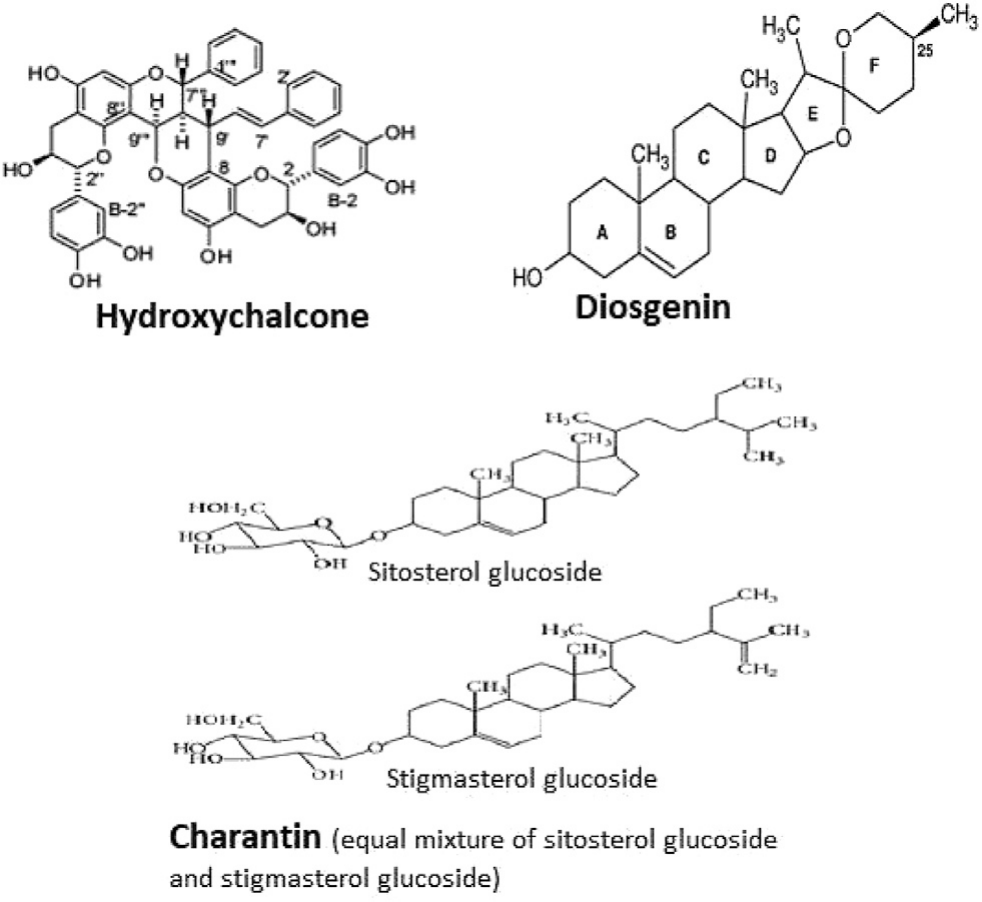
Structure of hydroxychalcone, diosgenin, and charantin.

Herbal drugs are prone to content variations in each harvest and geographical source and hence need serious efforts to be put in standardizing with respect to that of bioavailability and pharmacokinetic study of the herbal formulations. In the present study, three active phytoconstituents viz diosgenin, charantin, and hydroxychalcone were studied for their bioavailabilities and pharmacokinetic parameters in albino rats, as the scientific data regarding their bioavailabilities and pharmacokinetic parameters could not be traced (Figure 1).

## Materials and Methods

### Chemicals and Reagents

The seeds of *Trigonella foenum-graecum* L. and the bark of *Cinnamomum verum* J. Presl were purchased from the commercial supplier, batch number; 204480093, Sanjeevani Ayurvedic Stores, Angel park, shop number 4, Kalamboli, Navi Mumbai, Maharashtra, India. The fruits of *Momordica charantia* L. were purchased from the local market Kalamboli, Navi Mumbai, Maharashtra, India. Seeds of *T. foenum-graecum*, fresh fruits of *M. charantia*, bark of *C. verum* were authenticated by Dr. Harshad Pandit from Guru Nanak Khalsa College Matunga, Mumbai. The voucher specimen numbers rbs p 014060319 (*Trigonella foenum graecum* L.), rbs p 013960219 (*Momordica charantia* L.), rbs p 013980219 (*Cinnamomum verum* J. Presl), and rbs p 014040319 (*Piper nigrum* L.) are deposited, respectively. The active marker compounds diosgenin, charantin, and hydroxychalcone were isolated in the laboratory of Saraswathi Vidya Bhavan’s College of Pharmacy, Mumbai, India and were characterized using chromatographic and spectral techniques by SAIF (Sophisticated Analytical Instrumentation Facilities), IIT Bombay. Solvents viz. chloroform, glacial acetic acid, and methanol were of analytical reagent (AR) grade. All other reagents used were of laboratory reagent (LR) grade and were used without further purification. For chromatographic analysis the HPLC grade solvents were utilized.

### Isolation of Marker Compounds

#### Isolation of Steroidal Sapogenins (Diosgenin and Charantin)

Diosgenin and charantin are aglycone parts of the steroidal saponin obtained from methanolic extracts of seeds of *T. foenum-graecum* L. and fruits of *M charantia* L., respectively by suitably modifying the reported methods (Sonal and Pratima, 2015; Chen et al., 2017). In brief, the plant materials (50 gm) were defatted with petroleum ether 100 ml (60–80°C), followed by extraction of the marc with polar solvent like methanol (200 ml), to get the glycoside enriched extract. Aglycones of these glycosides were obtained by refluxing the methanolic extracts with 2 M HCl. The aglycones released in this process were then partitioned into the organic phase consisting of diethyl ether (100 ml). The organic phases were then saponified with 10 ml of 10% alcoholic potassium hydroxide to get the aglycone in almost pure form. The aglycones were then purified by recrystallization.

#### Isolation of Hydroxychalcone

The bark powder of *C. verum* J. Presl (100 gm) was extracted in acetone (150 ml) at 50°C for 1.5 h. The extract was concentrated at the reduced pressure and the precipitate formed in resulting solution was dissolved using small amount of methanol. The methanolic extract was then fractionated using Sephadex LH-20 column. The yield obtained from polyphenol hydroxychalcone was 0.32 gm % w/w, and as it was not adequate for the experiments, simulated reaction was carried out using cinnamaldehyde (2 gm) and catechin (1 gm) in 50 ml acetone. The reactants were heated at 100°C for 50 mins to get the product having structurally similar polyphenolic present in the cinnamon bark (Tanaka et al., 2008). The synthesized product and the isolated marker compound were confirmed for their identity using spectral studies.

Characterization of the isolated marker compounds was done using TLC, UV-visible spectroscopy, NMR, HRLMS, and elemental analysis and comparing the data with the respective reference standards.

### Formulation of Capsules

Two capsule formulations F1 and F2 (Table 1) were prepared by incorporating the spray dried hydroalcoholic extracts of seeds of *T. foenum-graecum*, fresh fruits of *M. charantia*, and the bark of *C. zeylanicum*. The F2 formulation contained 1.5 mg of piperine. The extracts utilized for formulations were standardized for the content of diosgenin, charantin, and hydroxychalcone using the HPTLC method (Table 2).

**TABLE 1:** Composition of the herbal formulations developed in laboratory.

Sr no	Ingredients	Composition per capsule	
		Formulation 1	Formulation 2 (mg)
1	*Trigonella foenum-graecum* extract	42.8 mg	42.8
2	*Momordica charantia* extract	21.4 mg	21.4
3	*Cinnamomum zeylanicum* extract	10.71 mg	10.71
4	70% v/v aqueous ethanol solution	0.11 ml	0.11
5	Microcrystalline cellulose	51 mg	51
6	Piperine	—	1.5

**TABLE 2:** HPTLC conditions.

Application mode	Camag Linomat V using 100 µl Hamilton syringe
Development mode	CAMAG twin trough chamber
Plate material	Silica gel 60F254 pre-coated HPTLC plates
Application bandwidth	6 mm
Developing solvent	Chloroform: glacial acetic acid: methanol: water (4:3:2:1) v/v
Chamber saturation time	10 min
Developing distance	90 min
Development time	20 min
Detecting wave length	342 nm
Lamp	UV
Scanner	CAMAG TLC scanner II
Integrator	WIN CATS V 1.2.2

The dose for each extract was selected based upon the content of these extracts in the marketed formulation quanto diab-forte (*T foenum-graecum* extract 400 mg, *M charantia* extract 200 mg, and *C zeylanicum* extract 100 mg, respectively), which is utilized for the treatment of diabetes. The piperine content of 1.5 mg is added owing to the amount of the piperine utilized in the herbal formulations (Wadhwa et al., 2014; Mhaske et al., 2018).

The preparation of extracts for formulation of the capsules is described below.

#### Preparation of Extracts

In case of *T. foenum-graecum* L, the dried seeds were powdered and defatted using petroleum ether (100 ml) for 1 h, followed by extraction of the marc (50 gm), by refluxing it using 70% v/v hydroalcoholic solution. The extract (100 ml) was concentrated to 10 ml and then spray dried (Yield 4 gm).

For preparation of extract of fruits of *M. charantia* L, 50 gm of fresh fruits were grinded in the mixer and then it was extracted using 70% v/v hydroalcoholic solution (100 ml) for 3 h. The extract (150 ml) was concentrated to 10 ml, followed by spray drying (Yield 3 gm).

Extraction of cinnamon bark (50 gm) was carried out by refluxing the powder using 70% v/v hydroalcoholic solution, followed by reducing the extract (100 ml) to 10 ml followed by spray drying (Yield 7 gm).

#### Determination of Content of Marker Compounds

The contents of diosgenin, charantin, and hydroxychalcone were determined from spray dried extracts of *T. foenum graecum* L*, M. charantia* L*,* and *C. verum J. Presl*, respectively using the HPTLC method. The optimized chromatographic conditions are given in (Table 2).

The calibration curves were prepared for individual marker compounds in the range of 0.4 to 1.4 μg/ml.

The samples for analysis were prepared by dissolving 10 mg of individual spray dried extract in 10 ml of methanol to give the stock solution of 1 mg/ml (Solution A). 1 ml of Solution A was diluted to 10 ml with methanol to get a solution of 100 μg/ml (Solution B). Further dilutions were made in methanol. The samples were loaded on HPTLC plates and developed the chromatograms using the conditions described in (Table 2). The contents of the marker compounds were determined by extrapolating from respective calibration curves (Table 3).

**TABLE 3:** Content of diosgenin, charantin, and hydroxychalcone in the 70% v/v ethanolic extracts of *T foenum-graecum*, *M charantia*, and *C zeylanicum*, respectively.

Names of marker compounds in 70% v/v hydroalcoholic extracts	Mean retention time ±SD (mins)	Mean content of markers per 10 mg of extract ±SD (mean percent w/w)
Diosgenin from *T foenum-graecum*	1.742 ± 0.04	0.11 ± 0.005
Charantin from *M charantia*	3.942 ± 0.02	0.045 ± 0.02
Hydroxychalcone from *C zeylanicum*	7.717 ± 0.06	0.024 ± 0.03

Results are presented as mean ± SD (n = 3).

#### Preparation and Evaluation of Formulations

The standardized spray dried extracts were mixed in the proportions indicated in Table 1 and granules of the same were prepared using the wet granulation method. The dough was formed using microcrystalline cellulose (Avicel) and 60% v/v, followed by addition of required quantity of talc, and then powder blend was passed through sieve number 10 to produce the granules. The granules were gently spread and dried at 45°C in oven for 3 hrs followed by sieving through sieve number 20 supported on 40 and added 10% w/w fines. The granules were then filled in hard gelatin capsules, followed by evaluation of capsules for the content uniformity through determination of content of the marker compounds using the HPTLC method.

##### Sample Preparation

Granules from 10 capsules were mixed, and a weight of the powder equivalent to 75 mg and was transferred to 100 ml volumetric flask. About 20 ml of methanol (HPLC grade) was added and sonicated for 15 mins or till dissolution of extracts. The volume was then made to the mark using methanol (HPLC grade) (Stock A). The stock solution A was filtered and 1 ml aliquot of the filtrate was further diluted to 100 ml using methanol (HPLC grade). The solutions were further filtered through 0.45 μ syringe membrane and loaded on HPTLC plate for development of chromatogram and analyzed for the content of respective markers.

### Bioanalytical Method Development and Validation

The bioanalytical method was developed for the study of the bioavailabilities and the pharmacokinetic parameters of the marker compounds viz. diosgenin, charantin, and hydroxychalcone.

#### Animals

All animal experiments were approved by the Institutional Animal Ethics Committee (IAEC) and were in accordance with the Committee for the Purpose of Control and Supervision of Experiments on Animals (CPCSEA registration number: SVBCP/IAEC/M ph/18-19/65). Healthy Wistar male rats weighing 180–220 gm were procured from registered breeder Bharat Serum Pvt. Ltd.; Mumbai, India. Experimental animals were maintained on standard pelleted laboratory animal feed and water ad libitum. Animals were maintained at 22 ± 2°C and 55 ± 5% relative humidity in the light controlled (12 h light/12 h dark) room.

#### Analysis of the Marker Compounds in Plasma

Plasma concentrations of active marker compounds were determined using validated high-performance thin layer chromatography (HPTLC) with the UV detection method.

##### Preparation of Plasma Sample

The plasma samples (150 µl) were deproteinized by adding mixture of methanol: dichloromethane (1:1), followed by vortex mixing at 3000 rpm for 5 min. The supernatant was separated and further extracted with about 1.5 ml of ethyl acetate by mixing on vortex mixer at 3000 rpm for 10 min. The ethyl acetate layer was then collected and evaporated to dryness, followed by reconstitution with mobile phase (200 µl) containing chloroform: glacial acetic acid: methanol: water ((4.0:3.0:2.0:1.0) v/v and 100 µl was applied on a HPTLC plate. The optimized HPTLC conditions for development and quantification of the constituents is given in Table 2.

##### Preparation of Standard Solutions

Stock solutions of diosgenin, charantin, and hydroxychalcone were prepared by weighing 10 mg of each reference standard and dissolving in methanol (50 ml), followed by making up the volume to 100 ml with methanol to get the concentration of each marker as 100 ppm. The solution was then suitably diluted and 100 µl of the solution of the marker compounds was spiked in 100 µl plasma to get the concentration and prepared the solutions with concentrations in the range of 0.4–1.4 μg/ml of each marker compound. These were then utilized for preparation of calibration curve. The HPTLC analysis of samples and the standard solutions were carried out using the parameter described (Table 2).

The retarding factors (Rf) values for diosgenin, charantin, and hydroxychalcone were found to be 0.72, 0.61, 0.30, respectively. The lower limit of quantification of the method was 0.33, 0.090, 0.0072 μg/ml, respectively and linearity in the calibration curve were demonstrated up to an upper limit of 1.11, 1.13, 1.12 μg/ml, respectively. Intra- and inter-day precision and accuracy were determined as per ICH guidelines.

#### Method Validation

The validation of the analytical method was executed as per “Guidance for industry: Bioanalytical Method Validation” from the United States Food and Drug Administration (Guidance for Industry, Bioanalytical Method Validation, 2001).

##### Calibration Curve

**FIGURE 2:**
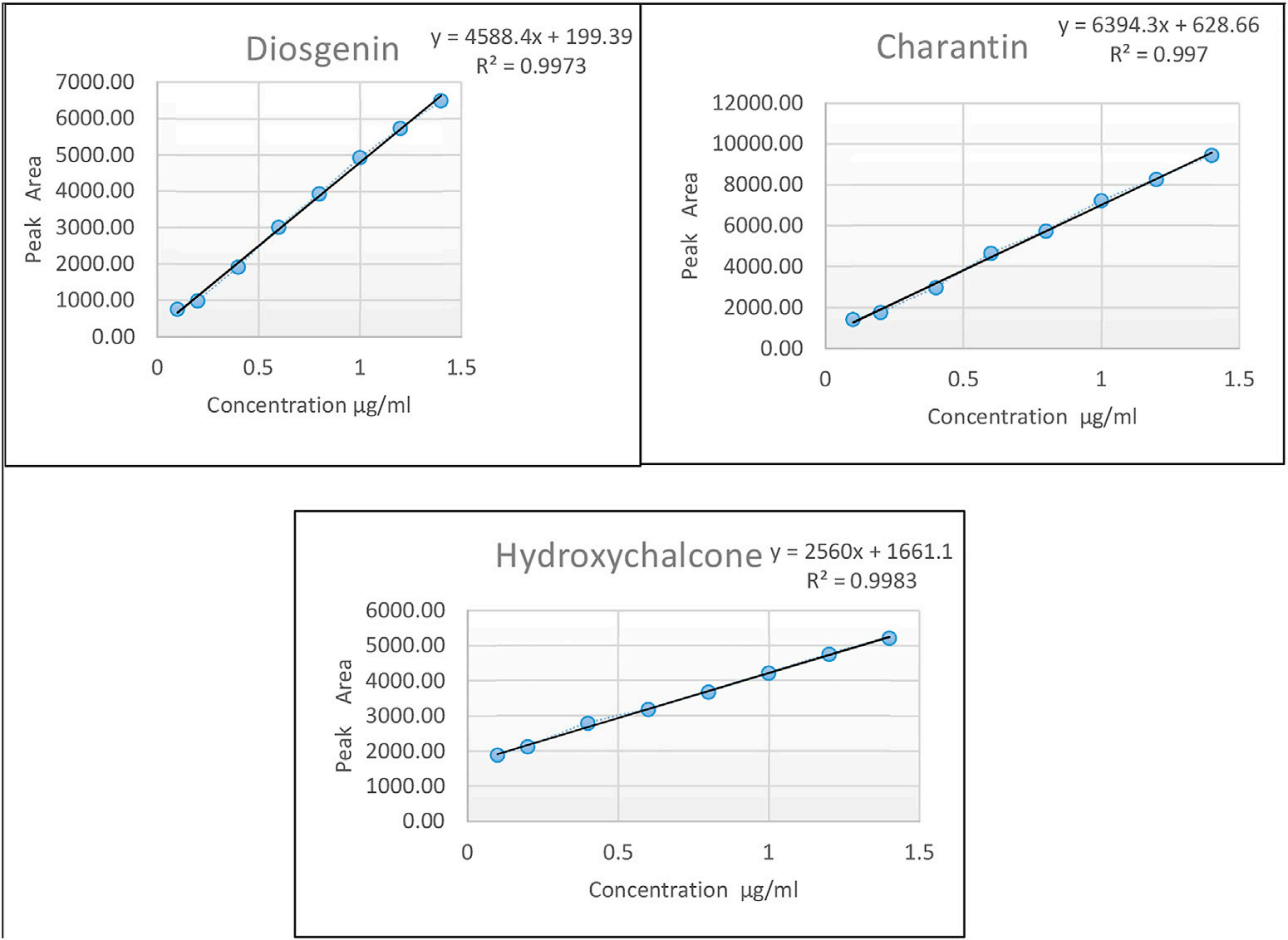
Calibration curves of the marker compounds viz., diosgenin, charantin, and hydroxychalcone, respectively.

For calibration curve, the stock solution containing all the three markers in the concentration of 100 ppm of each was diluted suitably and spiked in blank plasma to get different concentrations in the range of 0.4–1.4 μg/ml, followed by extraction as described in Section *Analysis of the marker compounds in Plasma*. The standard solution after extraction were applied on HPLTLC plates and developed using the conditions described in Table 2. The calibration curves were constructed individually by plotting peak area of diosgenin, charantin, and hydroxychalcone against the concentrations of the marker compounds using linear regression. The experiment was carried out in triplicate (Figure 2).

##### Selectivity

Selectivity of the analytical method was determined by recording the retardation factors of the marker compounds through HPTLC as described in Table 2. The blank plasma was also subjected to chromatographic analysis as described in Table 2. The third chromatogram was run by spiking the plasma with LLOQ (0.2 μg/ml) of marker solutions. The interference at the Rf values was determined by comparing the area response in the blank matrix against the mean response of the extracted LLOQ (0.2 μg/ml) samples. Also, interference at the Rf was evaluated by comparing the area response in the blank matrix against the mean response of extracted LLOQ (0.2 μg/ml) samples. The % interference was calculated by taking the ratio of “Area at the Rf of analyte to the Mean area (n = 5) at the Rf of analyte in the LLOQ.” The selectivity of the method was calculated using the following formula

$$\text{\% interference }=\frac{\text{area at Rf of analyte}}{\text{mean Area at Rf of analyte}}\times 100.$$

##### Accuracy and Precision

Accuracy studies were performed in terms of recoveries of hydroxychalcone, charantin, and diosgenin from spiked plasma. For this quality control samples, LQC–lower-concentration quality control sample (0.4 ppm) and MQC–medium concentration quality control samples (0.8 ppm) of all reference standard drugs were analyzed (n = 3), and % accuracy was calculated by comparing the average measured concentrations using the calibration curves to known concentrations.

The inter- and intra-day precision for the analytical method was determined by analyzing the quality control samples of all the marker compounds at LQC (0.4 PPM), MQC (0.8 PPM), and HQC (1.2 ppm) using the HPTLC method described above. For intra-day precision, RSD (Relative Standard Deviation) was calculated for analysis of the quality control samples in six replicates. In case of inter-day precision, RSD for analysis of the quality control samples carried out for three consecutive days was calculated. The analysis was carried out in six replicates.

##### Recovery and Stability

The extraction recoveries (ER) of diosgenin, charantin, and hydroxychalcone from plasma were determined at three different concentrations low-, middle-, and high-concentration range. Also unextracted dilution was prepared at concentration representing a 100% were applied/spotted and analyzed. The mean absolute % recovery was calculated by taking the ratio of “Mean peak area response of extracted samples at LQC/MQC/HQC” to the “Mean peak area response of unextracted samples at LQC/MQC/HQC.”

The mean absolute % recovery was calculated by taking the ratio of “Mean peak area response of extracted samples at MQC (0.8 μg/ml) level” to the “Mean peak area response of unextracted samples”. Overall recovery for analyte was calculated by taking the average of mean of absolute % recovery at LQC (0.4 μg/ml), MQC (0.8 μg/ml), and HQC (1.2 μg/ml) with SD and % CV for analyte are also reported.

Sample stability was determined by analyzing samples for quality control for analyte’s stock stability in solvent, refrigerator stock solution stability, freeze and thaw stability, and bench top stability.

### Pharmacokinetic Studies

The pharmacokinetic studies were carried out in male Wistar rats. Initially the absolute bioavailabilities of individual marker compounds were determined, followed by determination of bioavailability of the formulations F1 and F2 with respect to the marker compounds**.**

#### Determination of Absolute Bioavailability of Marker Compounds

Absolute bioavailabilities of marker compounds viz. diosgenin, charantin, and hydroxychalcone were determined in male Wistar rats. The animals weighing 300–350 gm were fasted overnight before the dosing with free access to drinking water throughout the experimental period. For the purpose of dosing all the three marker compounds viz, diosgenin, charantin, and hydroxychalcone were suspended in water to make the dose of 15 mg/kg per oral to be administrated to the groups of three rats. The animals were divided into two groups with six animals in each group. The first group received the marker compounds in the dose of 1.5 mg/kg i.v. *via* lateral tail vein, while the second group received the marker compounds in the dose of 15 mg/kg p.o. The rats were anesthetized using ether and blood samples were collected through puncturing the retro-orbital plexus at 0, 0.17, 0.5, 1, 2, 4, 6, 12, and 24 h time points. Blood samples were centrifuged to separate plasma and stored at −20°C until bioanalysis.

#### Determination of Bioavailability of Formulations

Bioavailabilities of the two formulations viz. F1 containing the standardized spray dried extracts of seeds of *T. foenum-graecum*, fresh fruits of *M. charantia*, and the bark of *C. verum*. The F2 containing the spray dried extracts as mentioned in F1 along with the piperine (1.5 mg) were determined by administrating the formulations in the dose of 75 mg/kg b.w. p.o. to the two groups (each group n = 6) of male Wistar rats weighing 300–350 gm, fasted overnight. For administration of the formulations, these were suspended in water with 2% w/v and Tween 80, and administrated through oral gavage. The blood samples were collected through puncturing the retro-orbital plexus at 0, 0.17, 0.5, 1, 2, 4, 6, 12, and 24 h time points. Blood samples were centrifuged to separate plasma and stored at −20°C until bioanalysis.

#### Pharmacokinetic Parameters (Jagannath et al., 2004; Michal et al., 2017)

The pharmacokinetic parameters were calculated for both the individual marker compounds as well as the formulations (F1 and F2).

Non-compartmental analysis of data was performed using statistical moment theory. The peak plasma concentration (C_max_) and the corresponding time (T_max_) were directly obtained from the raw data.

The area under the plasma concentration vs. time curve upto the last quantifiable point, AUC_(0-t)_, was calculated by the linear and log-linear trapezoidal rule summation:

$${\text{AUC}}_{\left(0-\text{t}\right)}\;=\sum \frac{{\text{C}}_{\text{0}}{+\text{C}}_{\text{t}}}{2}\times {\text{ T}}_{\text{t}}-{\text{ T}}_{\text{0}}.$$

C = Plasma drug concentration at time t.

T = Time required to attain C.

The AUC_(0-t)_ was extrapolated to infinity (i.e., AUC _(0–1)_) by adding the quotient of C_last_/K_el_,

$${\text{AUC}}_{\left(\text{t}-\infty \right)}\;=\;\frac{{\text{C}}_{\text{last}}}{{\text{K}}_{\text{el}}},$$

Where C_last_ represents the last measurable time concentration and K_el_ represents the elimination rate constant.

K_el_ was calculated by the linear regression of the log-transformed concentrations of the last three data points in the terminal phase.

$${\text{K}}_{\text{el}}\;=\;-\text{slope × }2\text{.}303\text{.}$$

Slope was obtained from log-transformed concentrations of the last three data points in the terminal phase.

The half-life of the elimination phase was obtained from using the relationship,

$${\text{t }}_{1/2\;}=\;\frac{\text{0}\text{.}693}{{\text{K}}_{\text{el}}}.$$

Clearance was calculated using the relationship Cl = Vd × K_el_

Vd = Apparent volume of distribution

K_el_ = Elimination rate constant

Apparent volume of distribution was calculated using relationship

$$\text{Vd }=\;\frac{\text{Dose Administered}}{\text{Plasma concentration}}.$$

Absolute oral bioavailability (F) was calculated using relationship F = {[AUC _(oral)_/AUC _(iv)_] × [Dose _(iv)_/Dose _(oral)_]} × 100.

#### Statistical Analysis

The results are expressed as mean ± standard deviation (SD). Data were statistically analyzed using Student’s t test. A *p* value of less than 0.05 was considered as statistically significant.

## Results and Discussion

### Isolation and Characterization of Marker Compounds

Isolation of marker compounds viz, diosgenin, charantin, and hydroxychalcone were done as per the procedure given in Section *Isolation of Marker compounds*. Identity of the isolated marker compounds was determined using chromatographic and spectral studies. The spectroscopic data of the marker compounds were compared with the standard marker compounds for the confirmation of the structures.

### Formulation of Capsule

For formulation of capsules, the content of diosgenin, charantin, and hydroxychalcone were determined in the spray dried extracts of *T foenum graecum* L*, M charantia* L, and *C verum* J. Presl, respectively using the HPTLC method.

#### Standardization of Extracts

The extracted samples were loaded on HPTLC plates and developed the chromatograms using the conditions described in (Table 2). The contents of the marker compounds were determined by extrapolating from respective calibration curves.

These standardized extracts were then utilized for preparation of polyherbal formulation.

#### Evaluation of Polyherbal Formulation

The evaluation of capsules was done for the content uniformity through determination of content of the marker compounds using the HPTLC method; results are represented in Table 4.

**TABLE 4:** Contents of diosgenin, charantin, and hydroxychalcone in polyherbal formulation determined using the optimized HPTLC method.

Name of the hydroalcoholic extracts present in formulation	Mean. Wt. of each capsule (n = 3)	Mean retention time (n = 3)	Mean content of markers (mg) in the formulation 75 mg (n = 3)	Mean % w/w content (n = 3)
*T foenum-graecum,* (D)	**197.52 ± 0.07**	1.742 ± 0.04	1.0280 ± 0.02	0.95 ± 0.01
*M charantia,* (CH)		3.942 ± 0.02	0.5240 ± 0.01	0.4 ± 0.01
*C zeylanicum,* (HC)		7.717 ± 0.06	0.0822 ± 0.005	0.079 ± 0.002

Results are expressed as mean ± SD (n = 3). D: Diosgenin, CH: Charantin HC: Hydroxychalcone.

### Validation of Bioanalytical Method

The validation was executed as per “Guidance for industry: Bioanalytical Method Validation” from the United States Food and Drug Administration (Guidance for Industry, Bioanalytical Method Validation, 2001). The results of validation parameters are given as follows.

#### Selectivity

**FIGURE 3:**
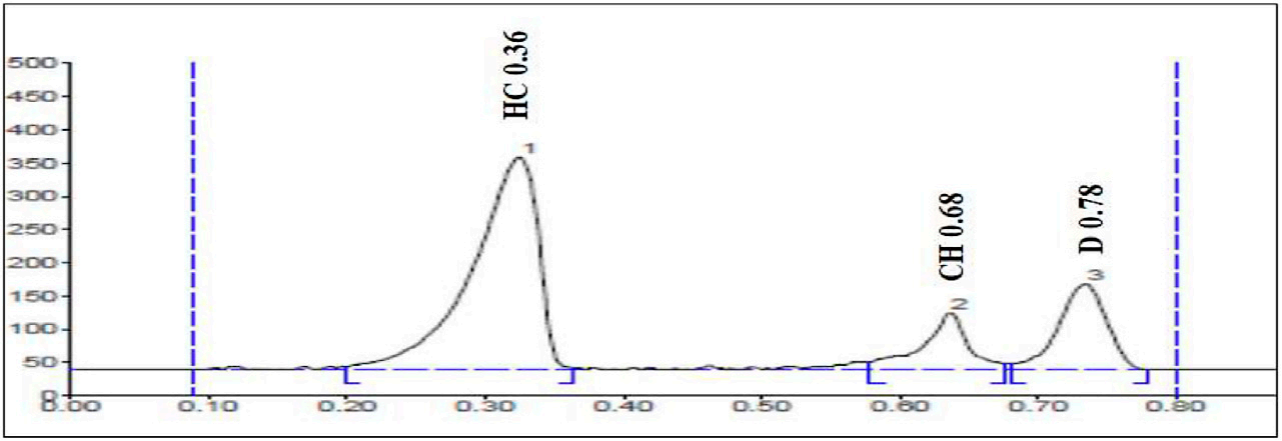
Chromatogram of plasma sample spiked with the markers HC (Hydroxychalcone), CH (Charantin), and D (Diosgenin) in the concentration of 0.2 μg/ml.

The selectivity of the method was evaluated by analyzing blank plasma samples prior to spiking. The representative chromatograms of the plasma sample spiked at 0.2 μg/ml of all the three marker compounds and compared with the blank plasma sample. The results presented in Figure 3 indicate that the chromatogram of the marker compounds was free of interfering peaks at the Rf values of diosgenin (D), charantin (CH), and hydroxychalcone (HC). Thus, it indicated the method selectivity Table 5.

**TABLE 5:** Selectivity study of diosgenin, charantin, and hydroxychalcone spiked at 0.2 μg/ml in rat plasma.

Marker compounds	Parameters		
	Area	SD	% Interference
Diosgenin (0.2 μg/ml)	2035.38	29.25	1.43
Charantin (0.2 μg/ml)	1157.03	20.58	1.77
Hydroxychalcone (0.2 μg/ml)	2465.03	40.78	1.65

#### Linearity of Calibration Curves

Linearity for the calibration curve of each marker compound was observed in the range of 0.4–1.4 µg/ml with the regression equations for the marker compounds as given below: the regression equation for diosgenin was Y = 1527.8 X + 65.653 (*R* ^2^ = 0.9955); the regression equation for charantin was Y = 2139.3 X + 203.2 (*R* ^2^ = 0.9953); the regression equation for hydroxychalcone was Y = 811.26 X + 599.29 (*R* ^2^ = 0.9952),

Where X was the concentrations of analytes in rat plasma and Y was the peak areas.

#### Precision

Precision studies were performed for inter-day and intra-day variation in developed method by applying quality control samples through HPTLC analysis using optimized chromatographic conditions, as given in Table 2. The % RSD values of the results of determination of concentrations at LQC, MQC, andHQC levels for hydroxychalcone, charantin, and diosgenin are found within the limit that is lower than 10% as shown in the Table 6, and the accuracy was within 85–120% for samples for quality control. The results demonstrated that the method qualifies the precision and accuracy criteria.

**TABLE 6:** Precision of diosgenin, charantin, and hydroxychalcone in rat plasma.

Marker compounds	Concentration (µg/ml)	Intra-day assay	Inter-day assay
		%RSD	%RSD
Diosgenin	0.4	0.91	0.90
	0.8	1.42	1.39
	1.2	0.44	1.00
Charantin	0.4	1.50	0.77
	0.8	0.51	1.50
	1.2	0.21	1.15
Hydroxychalcone	0.4	0.91	1.06
	0.8	0.49	0.33
	1.2	1.25	0.73

(n = 3).

#### Extraction Recovery and Stability

The mean % recoveries for diosgenin, charantin, and hydroxychalcone were found to be 94.25, 88.02, and 93.70%, respectively, at all three levels of quality control samples.

The percent recovery of diosgenin, charantin, and hydroxychalcone are observed within the acceptance criteria of 85–120%, indicates the accuracy of the HPTLC method utilized (ICH Guideline M10 On Bioanalytical Method Validation, 2019).

The stability experiments were carried out to evaluate the degradations of the marker compounds under different experimental conditions. The results presented in Table 7 indicate that there is no significant degradation of diosgenin, charantin, and hydroxychalcone in plasma under different experimental conditions.

**TABLE 7:** Stability data for diosgenin, charantin, and hydroxychalcone in rat plasma.

Marker compounds	Concentration (µg/ml)	Room temperature standard stock solution stability (%)	Refrigerator standard stock solution stability (%)	Freeze thaw stability (%)	Short-term room temperature stability (%)
Diosgenin	0.4	85.18 ± 0.45	91.73 ± 0.46	87.06	98.64
	0.8	84.86 ± 1.23	90.24 ± 1.13	94.52	97.90
	1.2	85.03 ± 2.16	89.56 ± 2.21	97.86	98.25
Charantin	0.4	86.79 ± 2.99	85.79 ± 2.43	74.01	95.25
	0.8	86.02 ± 3.49	88.13 ± 2.13	88.12	90.25
	1.2	88.39 ± 1.52	90.14 ± 2.02	97.85	96.54
Hydroxychalcone	0.4	87.59 ± 2.33	90.03 ± 1.56	63.73	63.76
	0.8	85.15 ± 1.67	94.16 ± 1.32	70.63	79.32
	1.2	87.00 ± 1.23	91.23 ± 1.84	79.32	79.02

Results are presented as mean ± SD (n = 3).

As per the acceptance criteria, % ratio (Stability/comparison) should be within 85–115%; from the above results, it was concluded that all the marker compounds were within the acceptance criteria for stability study Table 7.

### Pharmacokinetic Study

#### Determination of Absolute Bioavailability of Marker Compounds

The above validated bioanalytical HPTLC method was successfully and completely applied to evaluate pharmacokinetics in male Wistar rats. Initially the bioavailability of marker compounds was determined by administrating the pure marker compounds individually to Wistar rats by oral (dose 15 mg/kg b.w per oral) and parenteral (dose 1.5 mg/kg b.w i.v.) routes, followed by determination of content of the same in plasma at different time points by the HPTLC method. The results of the absolute bioavailability studies of the individual marker compounds are presented in Table 8.

**TABLE 8:** Mean (±SD) pharmacokinetic parameters and absolute bioavailabilities for diosgenin, charantin, and hydroxychalcone in rats after a single dose administration.

Parameters	Diosgenin		Charantin		Hydroxychalcone	
	Oral single dose administration (15 mg/kg per oral) (µg/ml)	IV single-dose administration (1.5 mg/kg i.v.) (µg/ml)	Oral single-dose administration (15 mg/kg per oral) (µg/ml)	IV single-dose administration (1.5 mg/kg i.v.) (µg/ml)	Oral single-dose administration (15 mg/kg per oral) (µg/ml)	IV single-dose administration (1.5 mg/kg i.v.) (µg/ml)
K_el_ (h^−1^)	0.0917 ± 0.003	0.0875 ± 0.004	0.0832 ± 0.0005	0.0844 ± 0.001	0.1481 ± 0.001	0.1488 ± 0.001
t _1/2_ (h)	7.5638 ± 0.21	7.9300 ± 0.37	8.3328 ± 0.05	8.2090 ± 0.13	4.6806 ± 0.04	4.6565 ± 0.04
Vd (ml/kg)	—	2.5397 ± 0.57	—	1.4108 ± 0.001	—	0.2799 ± 0.0004
Cl (ml/h/kg)	2.5143 ± 0.09	0.2221 ± 0.05	5.9268 ± 0.31	0.1191 ± 0.002	0.4199 ± 0.01	0.0417 ± 0.0004
AUC ^0^_∞_ (µg.h/ml)	4.4753 ± 0.06	4.9575 ± 0.44	12.4872 ± 0.11	15.2678 ± 0.03	72.9944 ± 0.49	69.2514 ± 0.09
C_max_ (µg/ml)	0.5773 ± 0.012	0.6122 ± 0.1	0.7725 ± 0.009	1.0633 ± 0.001	5.4245 ± 0.2	5.3583 ± 0.002
T_max_ (h)	1	-	6	—	1	—
AUMC^0^_∞_ (µg.h^2^/ml)	36.700 ± 0.77	17.3937 ± 2.10	176.7245 ± 1.83	182.9438 ± 1.74	574.5756 ± 1.08	547.3199 ± 1.21
MRT (h)	8.2008 ± 0.15	9.9420 ± 0.90	14.1521 ± 0.07	11.9823 ± 0.10	7.8716 ± 0.04	7.9034 ± 0.01
Ka (h^−1^)	1.1 ± 0.10	—	0.4617 ± 0.02	—	1.5 ± 0.16	—
Absolute bioavailability (%)	9.0 ± 0.2		8.18 ± 0.36		10.54 ± 0.52	

Results are presented as mean ± SD (n = 6) (K_el_- elimination rate constant, t_1/2_-plasma half-life, Cl-drug clearance, AUC-area under curve, C_max_-maximum concentration of the drug, T_*max*_-the time taken by a substance to reach the C_max_, AUMC-A under the moment curve, MRT-mean residence time, and Ka-rate of absorption).

**FIGURE 4:**
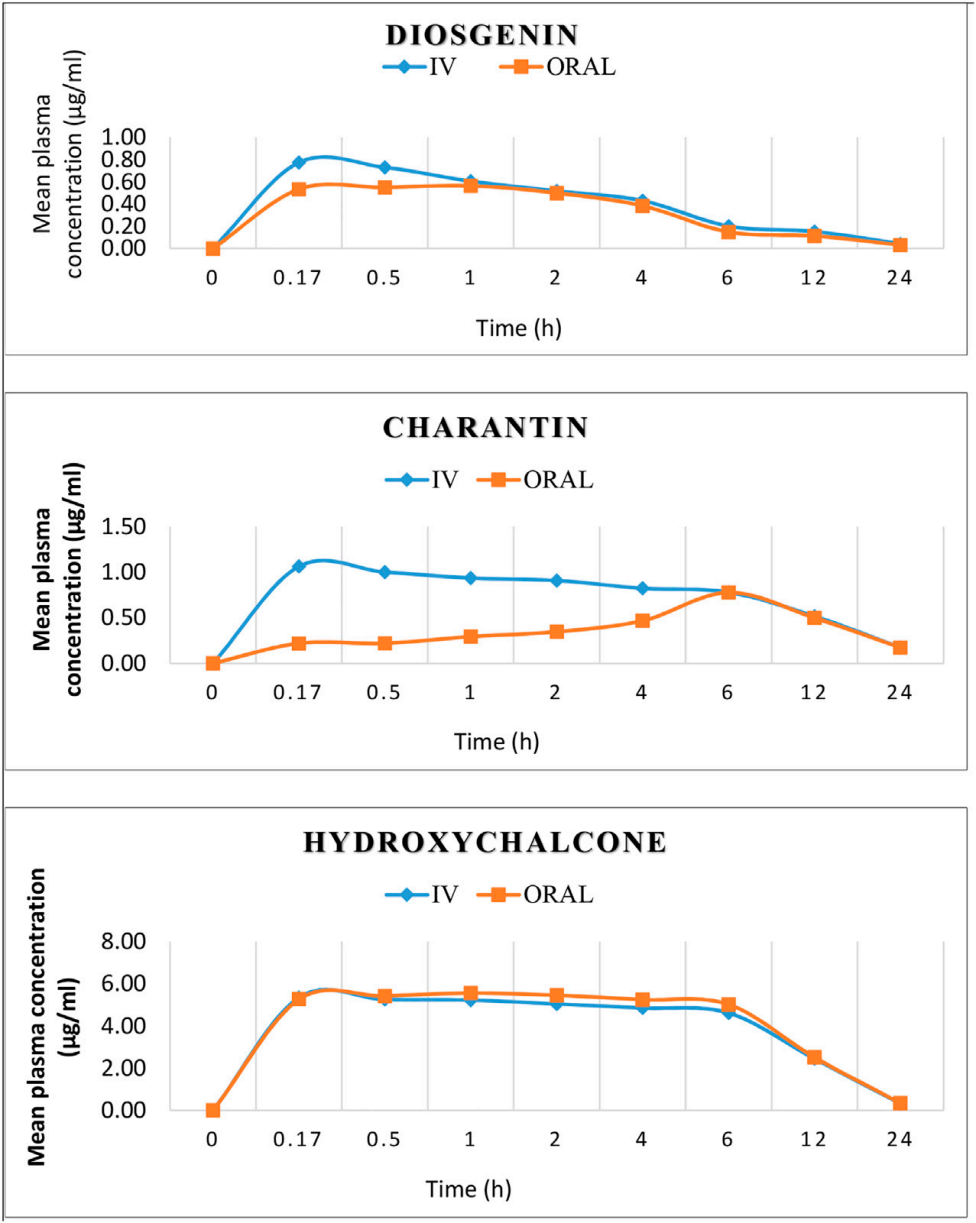
Mean concentration–time profiles of diosgenin, charantin, and hydroxychalcone after i.v. (1.5 mg/kg) and oral (15 mg/kg) administration.

The nature of the plasma curves following intravenous administration suggested a mono-exponential decline in the plasma concentration vs. time curves of the active marker compounds Figure 4.

Results presented in Table 8 reveal that the highest absolute bioavailability is obtained for hydroxychalcone (10.54%), when calculated by the dose normalization method probably due to polar nature, while the other two markers being steroidal, the bioavailability was between 8–9%.

Elimination rate constant (K_el_) indicates the rate at which a drug is removed from the body, which is found to be 0.09 and 0.08 h^−1^, 0.08 and 0.084 h^−1^, and 0.14 and 0.148 h^−1^ for i.v. and oral routes of administration for diosgenin, charantin, and hydroxychalcone, respectively. The similar K_el_ values for i.v. and oral routes of administration for marker compounds over the concentration range encountered in clinical settings indicate the first order elimination.

Plasma half-life (t_1/2_) indicates the time required for reducing the concentration of drug to the half of its concentration; it also indicates whether the accumulation of the drug occurs under a multiple dosage regimen and it is essential to decide on the appropriate dosing intervals. t_1/2_ was found to be 7.5638 and 7.9300 h i.v. and oral, respectively for diosgenin, 8.33 and 8.2 h for charantin, and 4.68 and 4.65 h for hydroxychalcone i.v. and oral, respectively, indicating there was no statistically significant difference in the values for intravenous as well as oral routes of administration due to first order elimination.

Volume of distribution (Vd) is theoretical volume that would contain the total amount of administrated drug at the same concentration in plasma. It represents the degree at which drug is distributed in the body tissue rather than plasma. Higher Vd indicates greater amount of tissue distribution and low protein binding. The highest volume of distribution was observed for diosgenin (2.5 ml/kg) followed by charantin (1.4 ml/kg) and least was observed for hydroxychalcone (0.27 ml/kg), indicating high protein binding of hydroxychalcone and ultimately low t_1/2_ and lower clearance of drug from the body. In case of diosgenin and charantin, as those are steroidal sapogenin, they have higher lipid solubility and have lower molecular weight compared to that of hydroxychalcone which is water soluble and having higher molecular weight; hence diosgenin and charantin have high volume of distribution and ultimately have high t_1/2_ values.

Drug clearance (Cl) is concerned with the rate at which a drug is removed from the body. It refers to the amount of the drug eliminated per unit time from the body. Clearance is related to the total drug concentration in the plasma (free + protein-bound) and not the free concentration. As per the results obtained, clearance for the hydroxychalcone was found to be 0.0417 ml/h/kg which is the least, followed by diosgenin (0.22 ml/h/kg) and charantin (0.11 ml/h/kg), indicating higher AUC for hydroxychalcone and the highest residence time of the drug in the systematic circulation and that slower will be the decline in plasma concentration of hydroxychalcone.

AUC represents the total drug exposure with respect to the time. Assuming the linear kinetics with elimination rate constant, one can show that AUC is equivalent to the total amount of drug observed in the body. AUC for the diosgenin was found to be 4.47 and 4.95 μg h/ml for i.v. and oral administration, respectively. AUC for charantin and hydroxychalcone was found to be 12.48 and 15.26 μg h/ml and 72.99 and 69.25 μg h/ml with i.v. and p.o. administrations, respectively. Higher AUC value of hydroxychalcone indicates more time hydroxychalcone will remain in the systematic circulation giving higher bioavailability.

T_max_ represents the time taken by a substance to reach the maximum concentration (C_max_) in the blood. Hydroxychalcone, being a weakly acidic compound due to presence of the phenolic groups, remains unionized at gastric pH and hence indicated very fast absorption after oral administration with short T_max_ of 1h. Charantin being nonpolar steroidal compound takes long time for its absorption which is revealed from the highest T_max_ value of 6 h. Although diosgenin is the nonpolar steroidal compound, it takes 1 h to reach the T_max_ due to higher number of OH groups in compound making it more polar as compared to the charantin. The results of pharmacokinetic parameters presented in Table 9, indicate that almost same C_max_ values of diosgenin, charantin, and hydroxychalcone, 0.57 and 0.61 μg/ml, 0.71 and 1.06 μg/ml, and 5.42 and 5.35 μg/ml, respectively, for i.v. and oral routes of administration were found.

**TABLE 9:** Relative bioavailabilities of diosgenin, charantin, and hydroxychalcone in the formulation without piperine (F1), formulation with piperine (F2), and oral pure drug administration.

Diosgenin:					
Formulations	AUC_0_^∞^ (µg.h/ml)	Dose (mg/kg p.o)	Relative bioavailability		
			Standard	Sample	Relative bioavailability
F2	1.9838	0.68	F2	F1	0.7346
Oral pure drug administration	4.4753	15	Oral pure drug administration	F1	9.7782
			Oral pure drug administration	F2	13.3113

Charantin:					
Formulations	AUC_0_^∞^ (µg.h/ml)	Dose (mg/kg p.o)
			Standard	Sample	Relative bioavailability
F2	1.6009	0.179	F2	F1	0.6836
Oral pure drug administration	12.4872	15	Oral pure drug administration	F1	10.7433
			Oral pure drug administration	F2	15.7160

Hydroxychalcone:					
Formulations	AUC_0_^∞^ (µg.h/ml)	Dose (mg/kg p.o)
			Standard	Sample	Relative bioavailability
F2	1.4294	0.0364	F2	F1	0.8523
Oral pure drug administration	72.994	15	Oral pure drug administration	F1	8.0701
			Oral pure drug administration	F2	9.4692

(F1-Formulation without piperine, F2-Formulation containing Piperine).

Mean residence time (MRT) is the average time the drug stays at the site of action. MRT for the marker compounds viz, diosgenin, charantin, and hydroxychalcone was found to be 8.20 and 9.94 h, 14.75 and 11.98 h, and 7.87 and 7.90 h for i.v. and oral routes of administration, respectively. MRT is inversely proportional to the AUC of the drug.

Rate of absorption (K_a_) determines the time required for the administrated drug to the reach an effective plasma concentration and may, thus, affect the onset of drug effect. K_a_ influences both the peak plasma concentration (C_max_) and time it takes to reach the peak (T_max_). Highest K_a_ value of 1.5 h ^−1^ was obtained in hydroxychalcone followed by diosgenin (1.1 h^−1^) and the least of case of charantin, 0.46 h^−1^. As the rate of absorption is 0.46 h^−1^, which is less compared to that of diosgenin and hydroxychalcone, it has higher T_max_ value of 6 h.

Bioavailability is the measurement of the rate and the extent to which drug reaches at the site of action. Absolute bioavailability compares the bioavailability of active drug in systemic circulation following non-intravenous administration, with the bioavailability of the same drug following intravenous administration. The absolute bioavailability of the drug, when administrated by extra vascular route is usually less than one (i.e., F <100%). In the present study, absolute bioavailability of diosgenin, charantin, and hydroxychalcone was found to be 9, 8.18, and 10.54%, respectively. Various physiological factors reduce the availability of the drugs prior to their entry into the systemic circulation. Higher absolute bioavailability for hydroxychalcone may be due to its higher tissue binding, high molecular weight, higher water solubility, and higher volume of distribution. On the other hand, diosgenin and charantin are the steroidal sapogenins having higher lipid solubility, hence have least volume of distribution, which affects their bioavailability.

#### Determination of Bioavailability of Formulations

The polyherbal formulations were prepared using the alcoholic extracts. The extracts contain several constituents that can influence the bioavailability and pharmacokinetic parameters of the marker compounds. Piperine is used in the current study as it is known for the enhancement of bioavailability of drug component from ancient time and in the traditional system of medicine Ayurveda (Chen et al., 2017; Mhaske et al., 2018).

**FIGURE 5:**
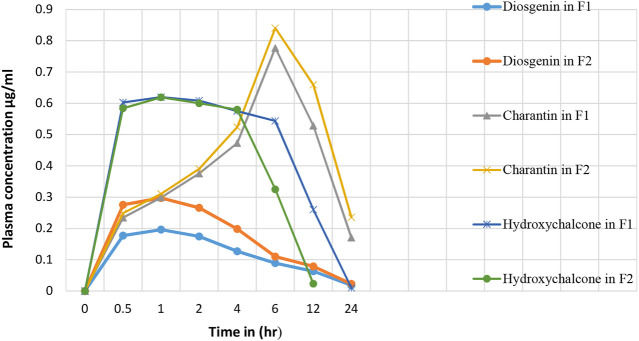
Effect of piperine in herbal formulation on the plasma drug concentration of diosgenin, charantin, and hydroxychalcone.

Results presented in Table 10 indicate the values of pharmacokinetic parameters obtained due to oral administration of the formulations F1 and F2 calculated with respect to the marker compounds viz. diosgenin, charantin, and hydroxychalcone. The elimination rate constants K_el_ values of the individual marker compounds in all the formulations were found to be similar when compared with the pure molecules diosgenin, charantin, and hydroxychalcone, respectively. Significant reduction (*p* < 0.01) in the clearance rate of all the three marker compounds is observed in formulation F2 as compared to the formulation F1, indicating availability of the marker compounds for activity due to inhibition of CYP enzymes by piperine Figure 5 (Volak et al., 2008).

**TABLE 10:** Effect of piperine on the pharmacokinetic parameters of the marker compounds viz, diosgenin, charantin, and hydroxychalcone after administration of the herbal formulations.

Parameters	Diosgenin		Charantin		Hydroxychalcone	
	F1 (75 mg/kg per oral) (µg/ml)	F2 (75 mg/kg per oral) µg/ml)	F1 (75 mg/kg per oral) (µg/ml)	F2 (75 mg/kg per oral) (µg/ml)	F1 (75 mg/kg per oral) (µg/ml)	F2 (75 mg/kg per oral) (µg/ml)
K_el_ (h^−1^)	0.0900 ± 0.004	0.0890 ± 0.001	0.0896 ± 0.0003	0.0844 ± 0.003	0.1450 ± 0.002	0.1412 ± 0.005
t _1/2_ (h)	7.7088 ± 0.32	7.7838 ± 0.117	7.7316 ± 0.025	8.2130 ± 0.25	4.7801 ± 0.08	4.9129 ± 0.169
Cl (mg/h/kg)	0.3456 ± 0.02	0.2199 ± 0.004	0.5553 ± 0.006	0.4548 ± 0.02	0.0685 ± 0.001	0.0350 ± 0.001
AUC _0_^∞^ (µg.h/ml)	1.9838 ± 0.02	2.7006 ± 0.006	1.6009 ± 0.034	2.3419 ± 0.05	1.4294 ± 0.01	1.6772 ± 0.003
C_max_ (µg/ml)	0.1962 ± 0.002	0.2975 ± 0.002	0.1008 ± 0.002	0.1506 ± 0.002	0.0954 ± 0.002	0.1517 ± 0.0001
T_max_ (h)	1	1	6	6	1	1
AUMC^0^_∞_	19.7230 ± 0.25	26.1961 ± 0.40	21.3542 ± 0.54	33.7730 ± 1.58	12.490 ± 0.20	12.7395 ± 0.27
MRT (h)	9.9420 ± 0.03	9.700 ± 0.15	13.3383 ± 0.05	14.4165 ± 0.38	8.7378 ± 0.08	7.5954 ± 0.15
Ka (h^−1^)	1.0 ± 0.02	1.1 ± 0.03	0.73 ± 0.04	0.41 ± 0.04	1.2 ± 0.09	1.5 ± 0.24

Results are presented as mean ± SD (n = 6), (K_el_-Elimination rate constant, t_1/2_- plasma half-life, Cl-drug clearance, AUC- area under curve, C_max_-maximum concentration of the drug, T_max_-the time taken by a substance to reach the C_max_, AUMC- A under the moment curve, MRT-mean residence time, and Ka-rate of absorption).

There is a significant increase (*p* < 0.01) in the AUC_0_^∞^ values of individual marker compounds in case of administration of the formulations F2 as compared to F1, indicating availability of significantly higher concentrations of the phytoconstituents for action. This is due to the bioenhancing effect of piperine through inhibition of CYP2D6 and CYP3A4 (ICH Guideline M10 on Bioanalytical Method Validation, 2019)_._

t _½_ represents the time required to reduce the concentration of the drug to be half of C_max_ value due to the elimination process. This value in turn also indicates the time for which the drug remains in the body. There was no significant difference in t_½_. The highest MRT values of 13.33 and 14.41 h were observed for charantin in F1 and F2, respectively.

Absorption rate constant Ka values for marker compound was found to be significantly increased (*p* < 0.05) for diosgenin and charantin in F2 as compared to F1, indicating more availability through improved absorption rate constant. In case of charantin, there was a decrease in the value of Ka of the formulation F2 as compared to the formulation F1, indicating increased rate of permeation due to piperine in F2.

Relative bioavailability measures the bioavailability of a formulation of certain drug when compared with another formulation of the same drug. Relative bioavailability is one of the measures used to assess bioequivalence between two drug products. From the results of bioavailabilities of the markers viz. diosgenin, charantin, and hydroxychalcone from the formulations F1 and F2, the relative bioavailabilities for these were calculated and the results are presented in Table 9.

Results presented in Table 9 indicate that the relative bioavailability of diosgenin in the formulation F1 when calculated with reference to F2 was found to be 0.73. When the relative bioavailability for F1 was compared to that of pure oral drug, it was found to be 9.77. Relative bioavailability for the formulation F2 when compared to the pure drug was found to be 13.31. Hence, it can be concluded that there is a 13-fold increase in the bioavailability of diosgenin due to presence of piperine.

As per the results for charantin, the relative bioavailability of the charantin in F1 when compared to that of F2 was found to be 0.68. Relative bioavailability of F1 when compared to that of the oral pure drug was found to be 10.74. At the same time, when relative bioavailability of F2 was compared to that of pure drug, it was found to be 15.71. Relative bioavailability of the formulation has been increased due to piperine.

The relative bioavailability of the hydroxychalcone in F1 when compared with that of F2 was found to be 0.85. When relative bioavailability of F1 was compared with that of oral pure drug, it was found to be 8.07. When F2 was compared with that of the pure drug, it was found to be 9.46. Hence, it was observed that there was an increase in the relative bioavailability of formulation due to presence of piperine.

Results of the relative bioavailability as presented in Table 10 clearly indicate that there was an increase in the bioavailability of the formulation due to incorporation of piperine. Piperine was known for the enhancement of bioavailability of the drug components from the ancient times. When relative bioavailability was studied, it was found that there was a 13- to 15-fold increase (*p* < 0.01) in the bioavailability of the marker compounds in the formulation F2 due to piperine.

## Conclusion

In conclusion, a bioanalytical HPTLC method has been developed and validated for the first time for evaluation of pharmacokinetics and absolute bioavailabilities of marker compounds diosgenin, charantin, and hydroxychalcone in single oral and intravenous administrated rats. The absolute bioavailabilities of marker compounds were found to be in the range of 8 to 10% which is increased by about 15% in formulation due to incorporation of piperine.

Further studies can be carried out at various dose levels and measures can be taken for improving the bioavailability.

## Data Availability

The original contributions presented in the study are included in the article/Supplementary Material; further inquiries can be directed to the corresponding author.
