# Supplementary material for: Pharmacokinetic Profile and Oral Bioavailability of Diosgenin, Charantin, and Hydroxychalcone From a Polyherbal Formulation
Source: Front Pharmacol. 2021 Apr 29;12:629272. doi: 10.3389/fphar.2021.629272 (PMC8117003; doi:10.3389/fphar.2021.629272)
Supplement: Supplementary file 1 [file DataSheet1.PDF]

Pharmacokinetic Profile and Oral bioavailability of Diosgenin, Charantin and Hydroxychalcone from a polyherbal formulation.

Ruchira Salunkhe[1*], Chhaya Gadgoli, Archana Naik and Nikita Patil.

[1] Saraswathi Vidya Bhavan's College of Pharmacy, Kalyan-Shill road, Dombivli East, 421204, Maharashtra, India.

*Corresponding author: ruchira.karpe@gmail.com

Supplementary data.

Authentication

Authentication is one of the essential parameters of standardization. Authenticity relates to proving that the material is true that is, it corresponds to the right identity. Authentication involves many parameters including gross morphology and microscopy. Thus, preliminary macroscopic and microscopic examination of the crude drug would enable the drug to be identified and also indicate whether the drug is likely to comply with other standard of quality and purity. Seeds of *T foenum-graecum*, fresh fruits of *M charantia*, bark of *C verum* were authenticated by Dr. Harshad Pandit from Guru Nanak Khalsa College Matunga, Mumbai. The voucher specimen numbers: rbs p 014060319 (*Trigonella foenum graecum*), rbs p 013960219 (*Momordica charantia*), rbs p 013980219 (*Cinnamomum zeylanicum*), rbs p 014040319 (*Piper nigrum*) are deposited respectively.

Authentication certificate of seeds of *Trigonella foenum-graecum L.*

HARSHAD M PANDIT, Ph.D. (Botany)
(Formerly Head and Associate Professor of Botany)
302 KAVERI
Bhardawadi Road
Adhere (West), Mumbai 400058
Maharashtra, India
Email: drhmpandit@gmail.com
Mobile: +919769955574

March 4, 2019

Ms. Ruchira Bapusaheb Salunke
M. Pharm. (Pharmacognosy & Phytochemistry) student
Saraswathi Vidya Bhavan's, College of Pharmacy
Sankara Nagar, Kalyan-Shill Road,
Sonarpada, M.I.D.C.
Dombivli (E)-421204.

Trigonella foenum-graecum Linn.

This is with reference to material in the form of seeds originating from the market. The seeds are rhomboid in shape with prominent diagonal notch with0.22 to 0.30 cm across and average size of 0.26 cm, yellowish brownish amber in colour. Internally the seed has columnar palisade with thick cuticle with cell with thickenings endosperm with aleurone grains, cotyledons with palisade and rudimentary spongy cells, aleurone and oil globules in cells. The seeds (**specimen #:rhs p 014060319**) are identified as ***Trigonella foenum-graecum*** Linn. belonging to family Fabaceae. It is commonly known as Fenugreek or Methi.

Yours sincerely,

Harshad M. Pandit (14909/88)

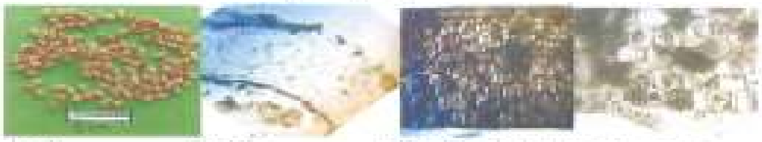

Seeds Cuticle Palisade, oil globules ,aleurone

Reference:

http://www.theplantlist.org/tpl1.1/record/ild-8021
http://www.tropicos.org/Name/13034390
http://specimens.kew.org/herbarium/K001122691

Authentication certificate of fresh fruits of *Momordica charantia*

HARSHAD M PANDIT, Ph.D. (Botany)
(Formerly Head and Associate Professor of Botany)
302 KAVERI
Bhardawadi Road
Adhere (West), Mumbai 400058
Maharashtra, India
Email: drhmpandit@gmail.com
Mobile: +919769955574

February 28th, 2019.

Ms. Ruchira Bapusaheb Salunke
M. Pharm. (Pharmacognosy & Phytochemistry) student
Saraswathi Vidya Bhavan's
College of Pharmacy
Sankara Nagar, Kalyan-Shill Road,
Sonarpada, M.I.D.C.
Dombivli (E)-421204.

Momordica charantia L.

This is with reference to material in the form of two entire fresh fruit for identification. The fruit are fusiform, pendulous, green, ribbed with many triangular tubercles approximately 18.50 cm, 22.20 cm in length and 3.35 cm, 9.83 cm broad. They are known as pepo (berry). The material (**specimen #: rbs p 013960219**) are the pepo of Momordica ***charantia*** L. synonym ***Momordica muricata*** Willd. ***Momordica indica*** L., ***Cucumis argyi*** H. Lév belonging to family Cucurbitaceae. It is commonly known as Karela, Bitter gourd, Bitter cucmber, Balsam pear.

Yours sincerely,

Harshad M. Pandit (14909/'85)

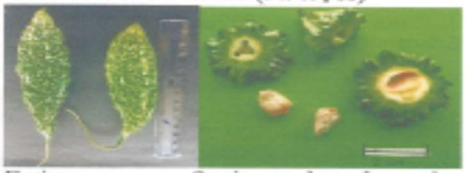

Entire pepo Section to show the seed

Reference:
http://www.tropicos.org/Name/9200010
http://specimens.kew.org/herbarium/K000742644

Authentication certificate of bark of *Cinnamomum verum* J. Presl

HARSHAD M PANDIT, Ph.D. (Botany)
(Formerly Head and Associate Professor of Botany)
302 KAVERI
Bhardawadi Road
Adhere (West), Mumbai 400058
Maharashtra, India
Email: drhmpandit@gmail.com
Mobile: +919769955574

February 28th, 2019.

Ms. Ruchira Bapusaheb Salunke
M. Pharm.(Pharmacognosy & Phytochemistry) student
Saraswathi Vidya Bhavan's, College of Pharmacy
Sankara Nagar, Kalyan-Shill Road,
Sonarpada, M.I.D.C.
Dombivli (E)-421204.

Cinnamomum zeylanicum Blume

This is with reference to dried bark material originating from market provided by you for identification. The bark is reddish brown to dark brown, thicker curling in both the sides and rough in surface and uneven. Internally the bark shows sclerenchyma with unequally thickened cells, bast cells, parenchyma with starch & crystals, typical thickened parenchyma, resin and oil cells. The material (**specimen #: rbs p 013980219**) is identified as the bark of ***Cinnamomum zeylanicum*** Blume synonym ***Cinnamomum verum*** J. Presl. ***Laurus cinnamomum*** L. ***Cinnamomum zeylanicum*** Breyn belonging to family Lauraceae. It is commonly as Darchini, Dalchini, Chadana, Tvak, Common cinnamon.

Yours sincerely,

Harshad M. Pandit (14909/85)

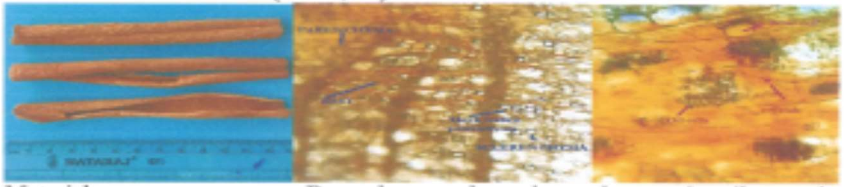

Material Parenchyma, sclerenchyma, bast, resin, oil, crystals

Reference:

http://specimens.kew.org/herbarium/K000778745
http://www.tropicos.org/Name/17800682?tab=synonyms
http://www.plantnames.unimelb.edu.au/Sorting/Cinnamomum.html

Authentication certificate of fruits of *Piper nigrum L.*

HARSHAD M PANDIT, Ph.D. (Botany)
(Formerly Head and Associate Professor of Botany)
302 KAVERI
Bhardawadi Road
Adhere (West), Mumbai 400058
Maharashtra, India
Email: drhmpandit@gmail.com
Mobile: +919769955574

March 4, 2019

Ms. Ruchira Bapusaheb Salunke
M. Pharm.(Pharmacognosy & Phytochemistry) student
Saraswathi Vidya Bhavan's, College of Pharmacy
Sankara Nagar, Kalyan-Shill Road,
Sonarpada, M.I.D.C.
Dombivli (E)-421204.

Piper nigrum Linn.

This is with reference to material originating from market provided by you for identification. The material is in the form of fruit. It is dried drupaceous fruit grayish black to black, hard, wrinkled approximately 0.43 to 0.52 cm with average size of 0.49 cm, aromatic with pungent taste. Internally epicarp of tubular stone cells, mesocarp parenchymatous oil cells and endocarp of peculiar stone cells, perisperm parenchymatous with oil globules and starch. The fruit or peppercorn (**specimen #: rbs p 014040319**) is identified as that of ***Piper nigrum*** Linn. synonym ***Piper aromaticum*** Lam. belonging to family Piperaceae. It is commonly known as Black pepper, Maricha, Kalimirch, Karimonaru, Kalimiri.

Yours sincerely

Harshad M. Pandit (14909/85)

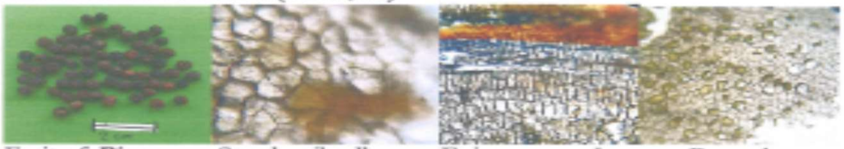

Fruit of ***Piper*** Starch, oil cells Epi, meso, endocarp Parenchyma, oil globules

Reference:
http://www.theplantlist.org/tpl1.1/record/kew-2569664
http://www.tropicos.org/Name/25000013
http://www.tropicos.org/Image/100532961

1 Characterization of the isolated marker compounds.

Identity of the isolated marker compounds was determined using chromatographic and spectral studies.

1.1 TLC analysis

Purity and partial characterization of the marker compounds was established by co-chromatographic TLC technique.

The results presented in Fig 1 indicated the isolated marker compounds viz.,

A) Diosgenin when co-chromatogramed with standard Diosgenin revealed that Rf of both the spots (standard and isolated) are coinciding and the compound give reaction with Anisaldehyde with development of green colour.

B) The two spots in Charantin corresponding to stigmasterol and sitosterol glucoside observed at Rf 0.9 and 0.5 respectively, and this were found to be corresponding with reference standard. The other spot corresponding to sitosterol as it is reported that Charantin is the equal mixture of stigmasterol and sitosterol.

C) The TLC analysis of the same presented in the Fig 1 indicates that the synthesized Hydroxychalcone and the isolated Hydroxychalcone Rf values are coinciding and both the compounds giving the reaction with $FeCl_3$ Indicating the presence of Hydroxychalcone.

D) TLC analysis using standard piperine was carried out and Fig 1 indicates the Rf values of both the spots are similar and the spot also yielded orange colour with Dragendroff's reagent indicating the presence of alkaloid.

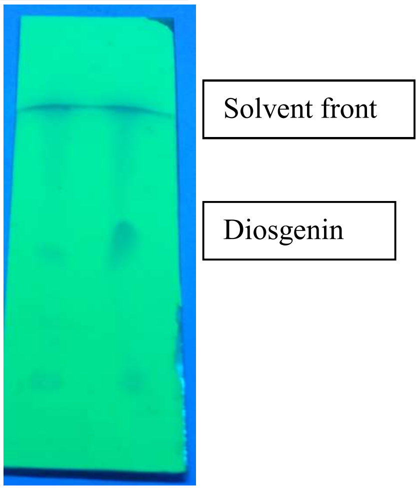

MP: Toluene: Ethylacetate: Methanol:

Formic acid (7:1.5:1:0.5)

Derivatizing reagent: Anisaldehyde sulphuric acid.

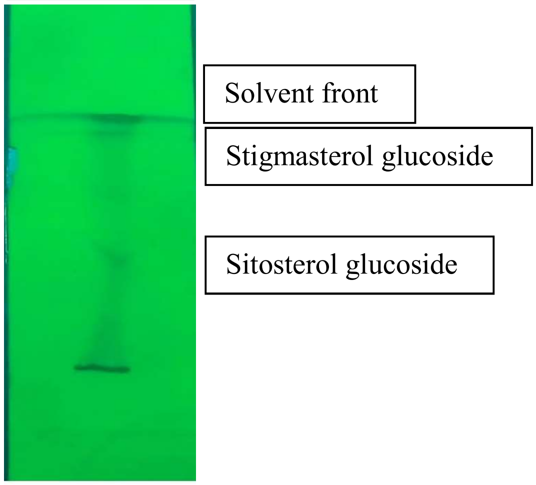

MP: Toluene: Ethylacetate: Methanol: Formic acid (7:1.5:1:0.5)

Derivatizing reagent: Anisaldehyde sulphuric acid.

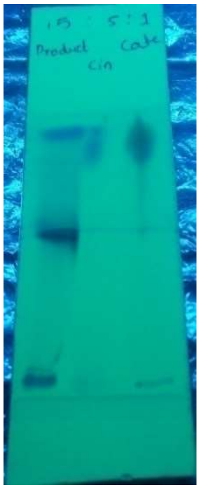

MP: Chloroform: Methanol: water (15:5:1)

Derivatizing reagent: Ferric chloride solution

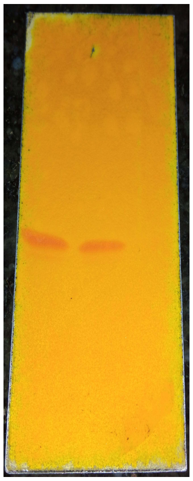

MP: Toluene: Ethyl acetate (7:3)

Derivatizing reagent: Dragendroff's reagent

Fig 1 TLC analysis of isolated marker compounds.

The TLC analysis gives preliminary information about identity of compounds; however, the spectral studies further help in confirmation of the compounds.

1.2 UV-visible spectrum of isolated compounds

UV-visible spectroscopy is the technique that readily allows one to determine the concentration of the substances, and to perform the quantitative analysis of all the molecules that absorbs ultraviolet and visible magnetic radiations. UV spectrum of any compound gives the λmax values which can be compared with standard compounds or data in the literature.

a) UV-visible spectrum of Diosgenin

UV-visible spectrum showed wavelength of maximum absorption at 232 nm (abs 0.831) as shown in Fig 2

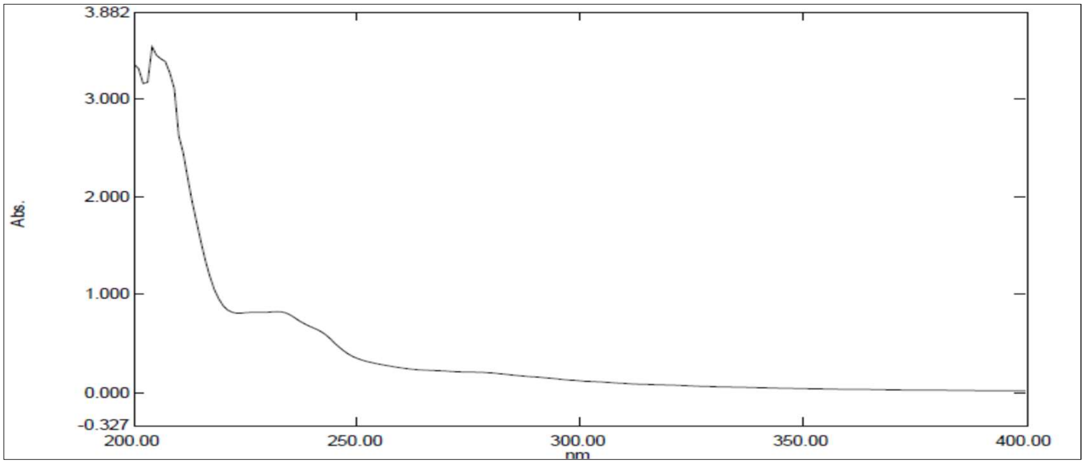

Fig 2 UV spectrum of Diosgenin

UV-visible spectrum of Charantin

UV-visible spectrum showed wavelength of maximum absorption at 207 nm (abs 0.451) as shown in Fig 3

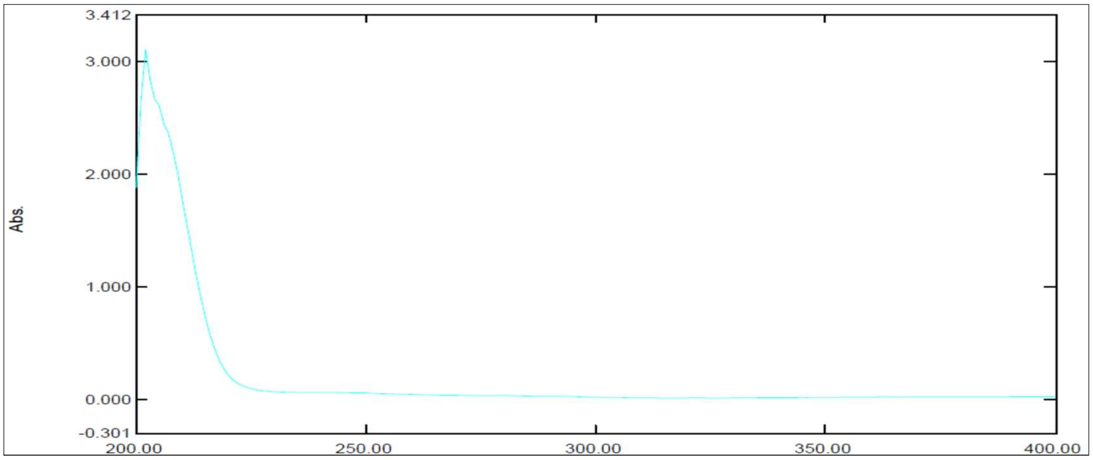

Fig 3 UV spectrum of Charantin

b) **UV-visible spectrum of Hydroxychalcone**

UV-visible spectrum showed wavelength of maximum absorption at 238 and 281 nm (abs 0.252 and 0.492 respectively) as shown in Fig 4

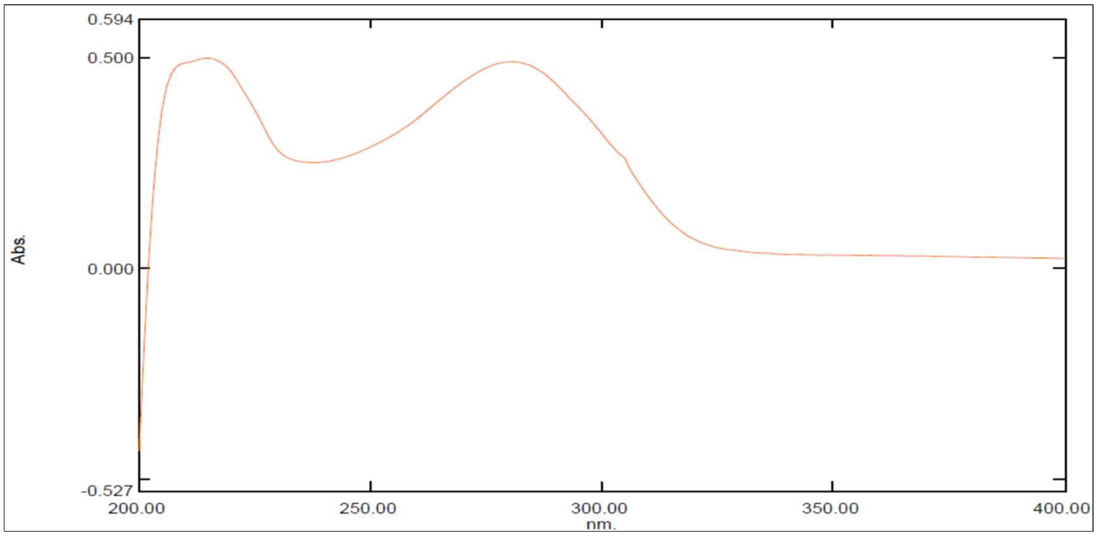

Fig 4 UV spectrum of Hydroxychalcone

Table 1 λmax values of the standard and isolated marker compounds.

Marker compound	λmax of Isolated compound	λmax of Standard compound
**Diosgenin**	232	224
**Charantin**	207	207
**Hydroxychalcone**	238nm and 281 nm	220nm and 300nm

The λmax values of the isolated marker compounds were found to be similar to that of the standard compounds.

1.3 NMR analysis of isolated compounds

Nuclear magnetic resonance analysis serves as the important tool for the detection of the local magnetic fields around the atom nuclei, thus giving access to the details of electronic structure of the molecules and its individual functional groups.

1) NMR analysis of Diosgenin

The NMR spectrum showed various peaks, which helped in characterization of the marker compound. The interpretation of NMR data was done with the help of standard values and correlated with the isolated diosgenin, the steroidal sapogenin present in *T foenum-greacum*.

a) [1] H NMR spectrum of isolated and standard Diosgenin:

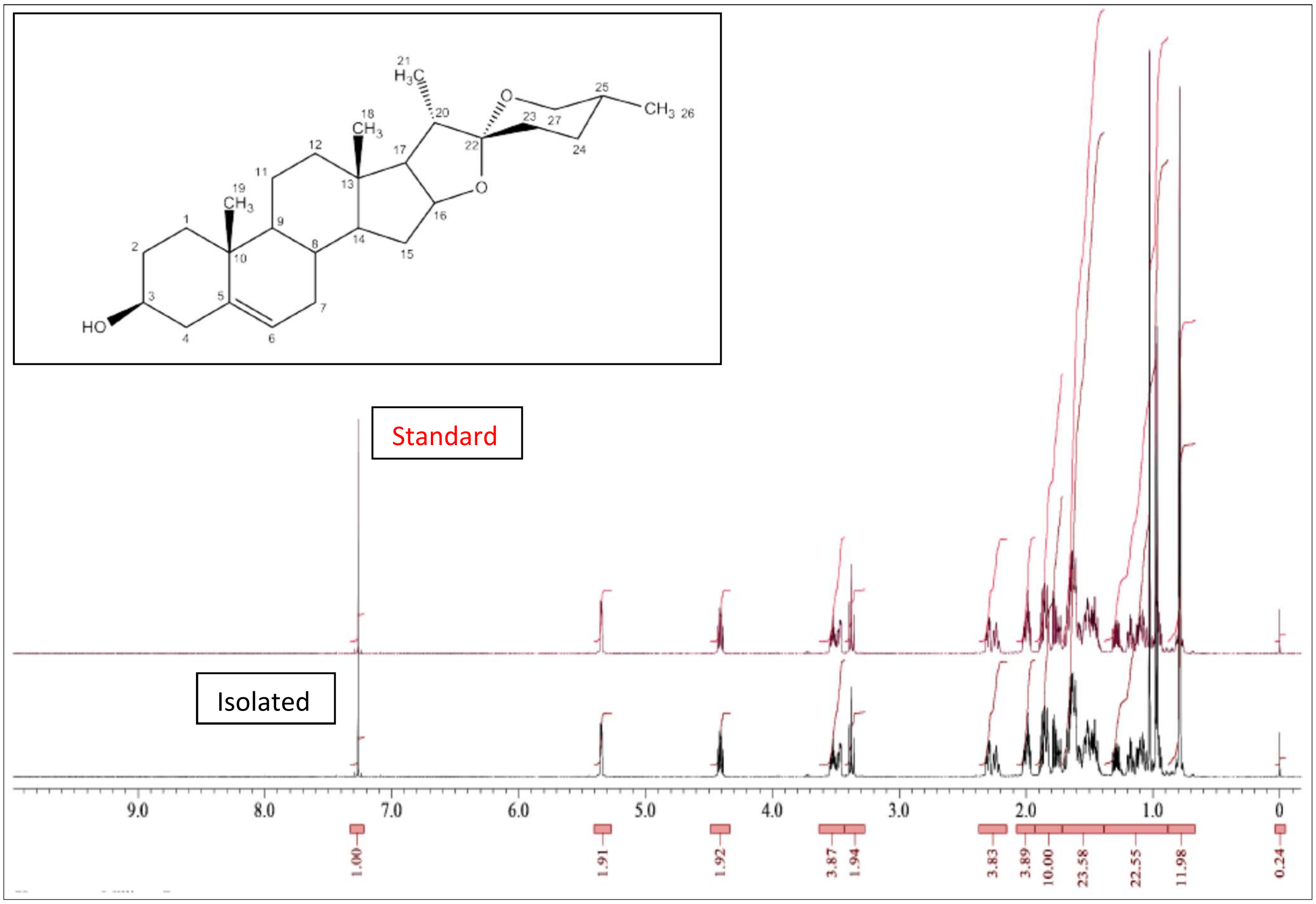

Fig 5 [1] H NMR spectrum overlay of isolated and standard Diosgenin

The assignment of signals of the compound in the spectra was obtained by the comparison with the corresponding signals of the standard Diosgenin.

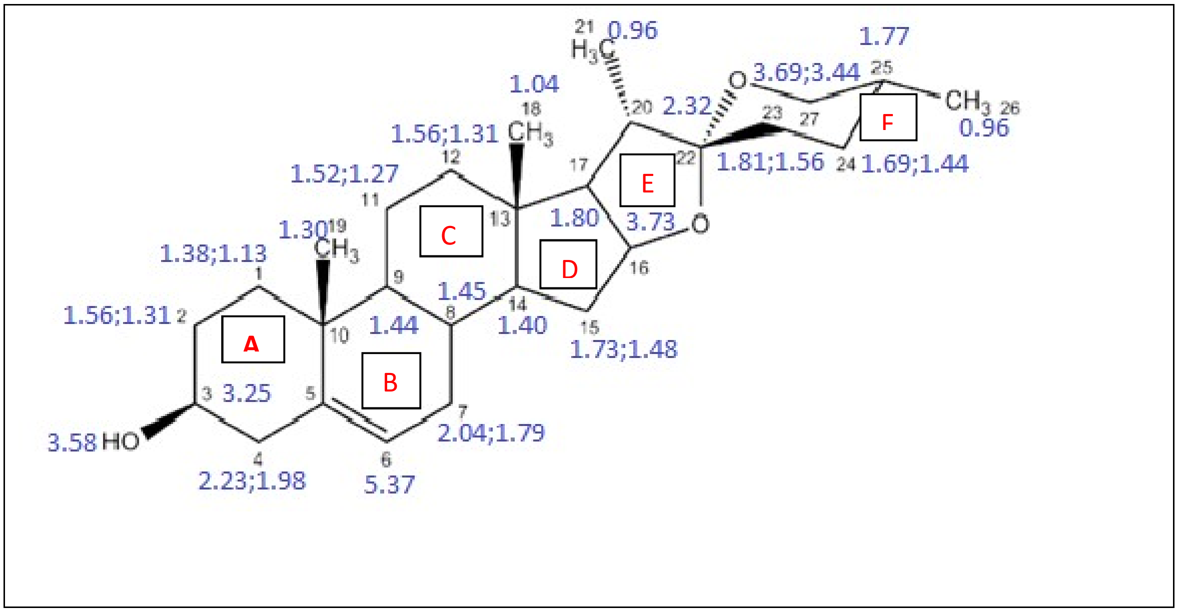

Fig 6 Structure of Diosgenin with chemical shift values

The following identical resonances were observed in the ^{1}H NMR spectra of standard and isolated Diosgenin.

Table 2 Correlation of ^{1}H NMR values of isolated Diosgenin with the standard Diosgenin (δ in ppm).

**Carbon**	**Standard diosgenin (ppm)**	**Isolated diosgenin (ppm)**	**Carbon**	**Standard diosgenin (ppm)**	**Isolated diosgenin (ppm)**
**1**	H-α1.05,H-β 1.82	H-α 1.03, H-β 1.85	**15**	H-α 1.96, H-β 1.26	H-α 1.96, H-β 1.27
**2**	H-α 1.81, H-β 1.48	H-α 1.82, H-β 1.47	**16**	4.38	4.39
**3**	3.51	3.52	**17**	1.78	1.76
**4**	H-α 2.22, H-β 2.27	H-α 2.22, H-β 2.26	**18**	0.76	0.78
**6**	5.30	5.36	**19**	1.04	-
**7**	H-α 1.52, H-β 1.95	H-α 1.52, H-β 1.96	**20**	1.85	1.86
**8**	1.63	1.62	**21**	0.92	0.94
**9**	0.94	0.94	**23**	H-α 1.58, H-β 1.68	H-β 1.65
**11**	1.51	1.52	**24**	H-α 1.64, H-β 1.43	H-α 1.64, H-β 1.43

**12**	H-α 1.17, H-β 1.72	H-α 1.17, H-β 1.73	**25**	1.61	1.61
**14**	1.08	1.08	**26**	H-α 3.38, H-β 3.36	H-α 3.38, H-β 3.37
**15**	H-α 1.96, H-β 1.26	H-α 1.96, H-β 1.27	**27**	0.76	0.76

In the 1H NMR spectrum, the signal arising at H-27 is the characteristic for the diosgenin.

The signal at H-6 indicated the presence of olefin proton and the methylene multiplet at the C-26.

The chemical shift values obtained for the standard diosgenin were similar to those of the isolated diosgenin with the presence of C-18 methyl group giving the signal at δ 0.79, C-21 methyl at δ 1.03, C-26α at δ3.38.

b) ^{13}C NMR spectrum of isolated and standard Diosgenin

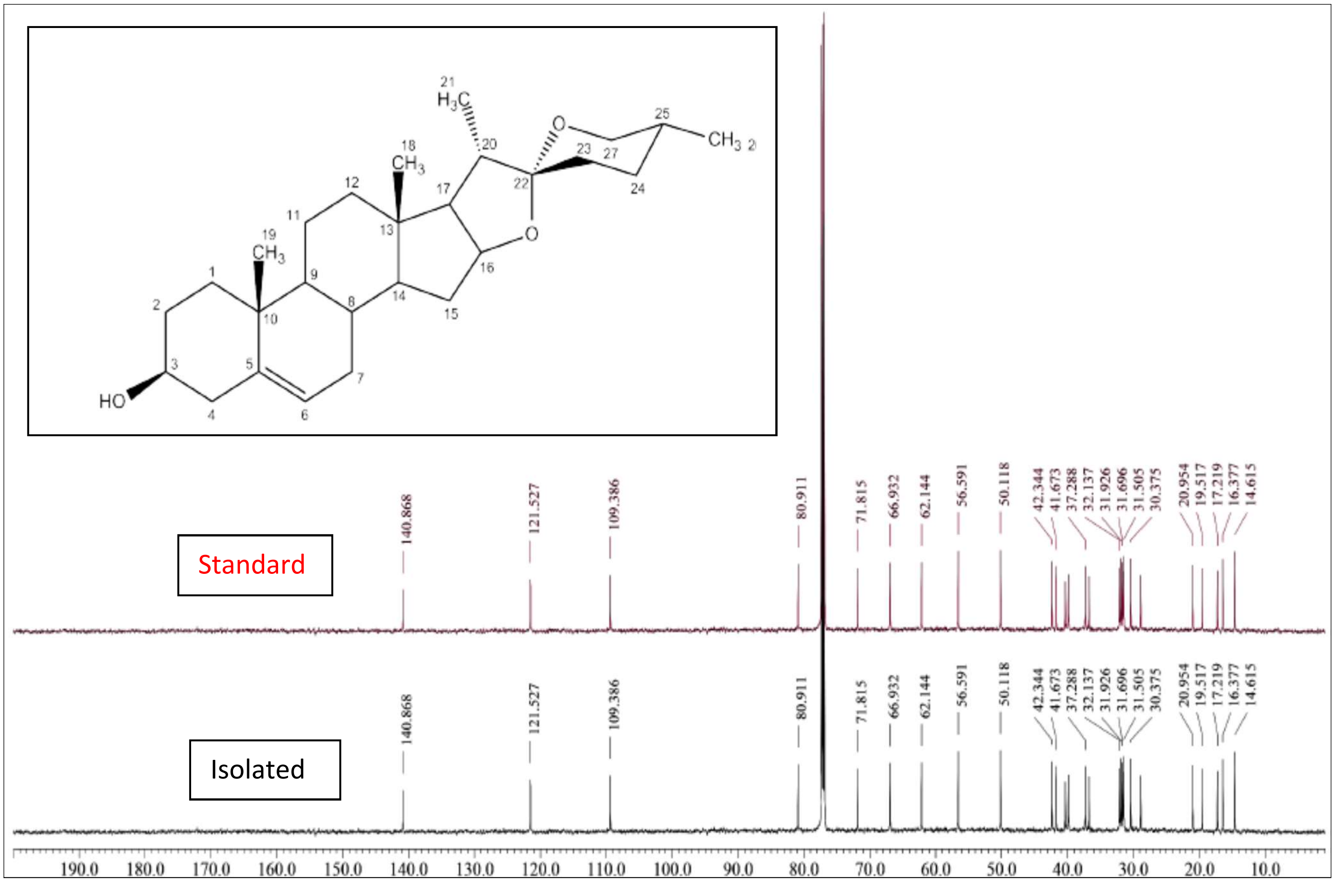

Fig 7 ^{13}C NMR spectrum of isolated and standard Diosgenin

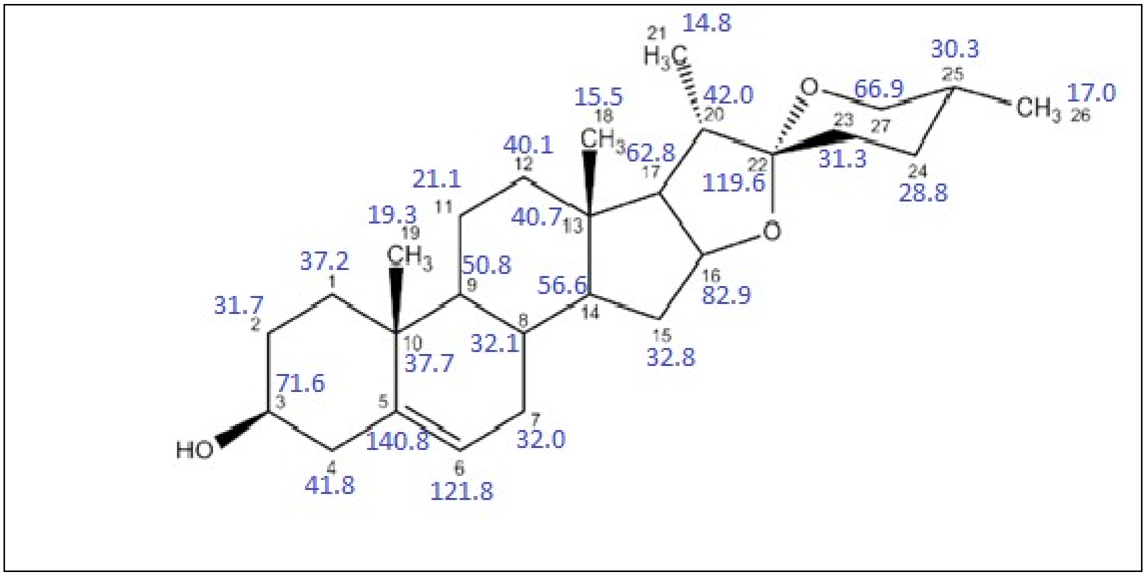

Fig 8 Structure of Diosgenin with chemical shift values

Table 3 Correlation of ^{13}C NMR values of isolated Diosgenin with the standard Diosgenin (ppm).

Carbon	Standard diosgenin (ppm)	Isolated diosgenin (ppm)	Carbon	Standard diosgenin (ppm)	Isolated diosgenin (ppm)
**1**	37.43	37.288	**15**	32.04	32.13
**2**	31.79	31.92	**16**	81.03	80.91
**3**	71.89	71.89	**17**	62.28	62.14
**4**	42.46	42.34	**18**	16.49	16.37
**5**	141.02	140.868	**19**	19.43	19.51
**6**	121.60	121.52	**20**	41.80	41.67
**7**	32.25	32.137	**21**	14.73	14.61
**8**	31.64	31.69	**22**	109.50	109.38

**9**	50.25	50.118	**23**	31.59	31.50
**10**	36.84	37.28	**24**	28.99	28.99
**11**	21.07	20.95	**25**	30.49	30.37
**12**	39.99	39.98	**26**	67.04	66.93
**13**	40.46	-	**27**	17.35	17.21
**14**	56.72	56.59			

The ^{1}H and ^{13}C NMR indicated that in the Diosgenin, 22-hydroxy has been converted into 22-methoxy on refluxing with methanol. Hydrolysis had led to the transformation the furostanal-26-O-glucoside to furostane sapogenin (Diosgenin).

2) NMR analysis of Hydroxychalcone

Nuclear magnetic resonance analysis serves as an important tool for detection of the polyphenolic compounds in C zeylanicum. The NMR spectrum showed various peaks, which helped in characterization of the marker compound Hydroxychalcon**e.** The interpretation of NMR data was done with the help of Isolated Hydroxychalcone values and correlated with synthesized Hydroxychalcone, the chalcone present in *C zeylanicum*.

a) [1] H NMR spectrum of isolated and standard Hydroxychalcone

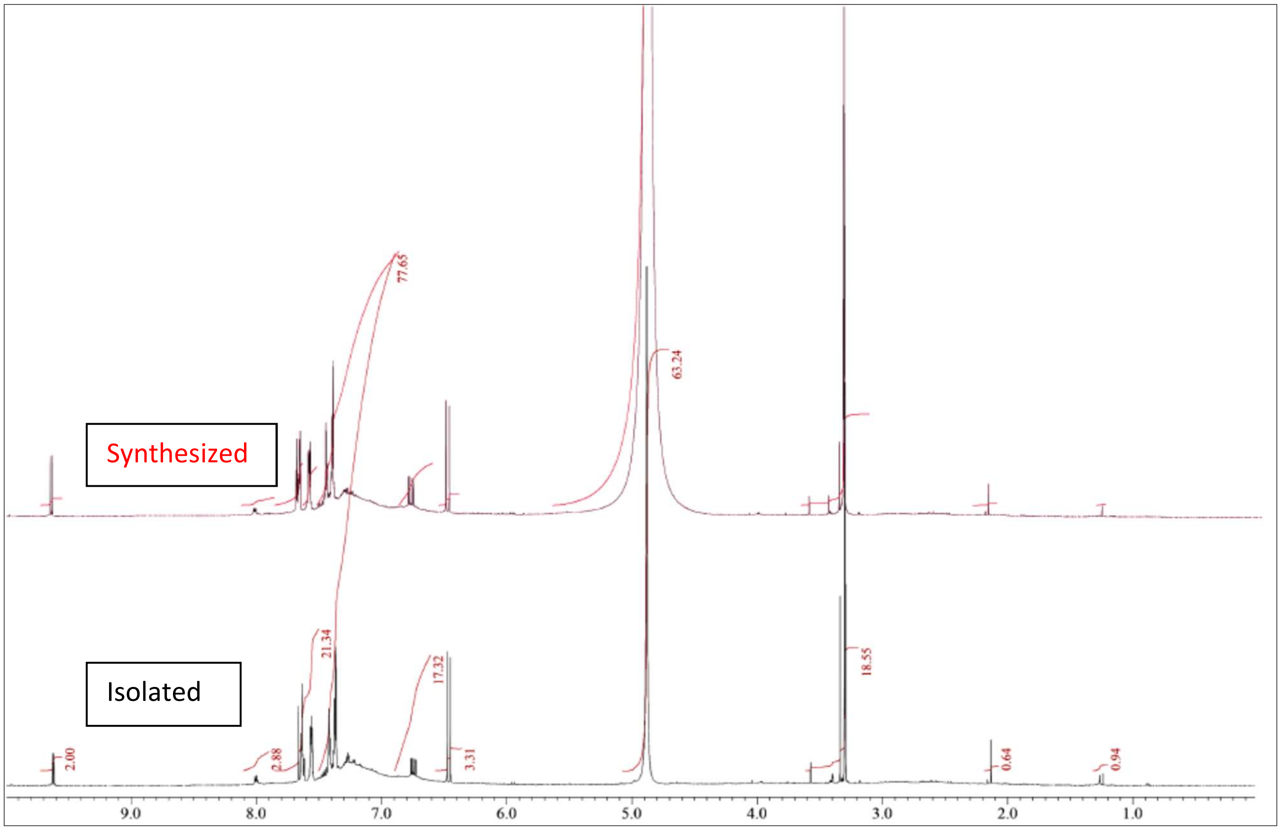

Fig.9 [1] H NMR spectrum of isolated and synthesized Hydroxychalcone

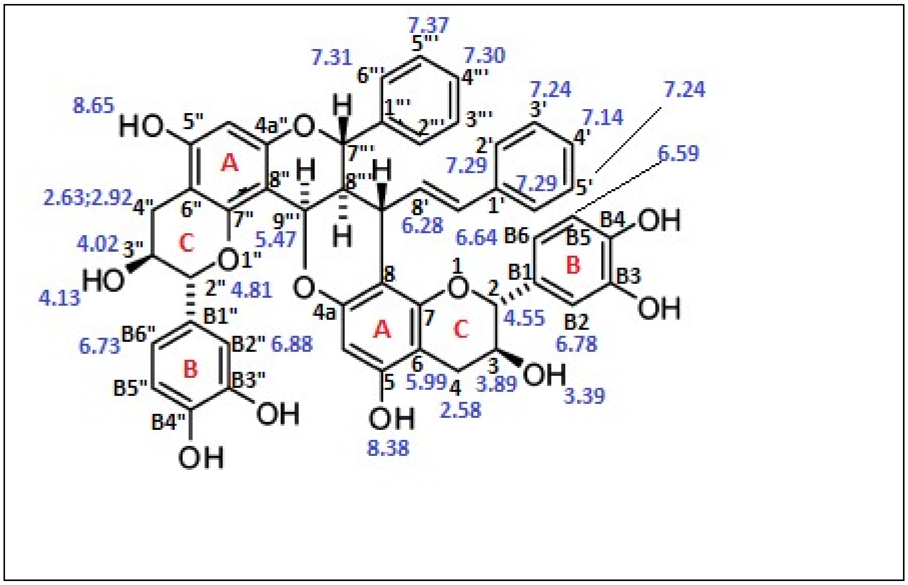

Fig 10 Structure of the isolated Hydroxychalcone.

Table 4 Correlation of ^{1}H NMR values of isolated Hydroxychalcone with the synthesized Hydroxychalcone (ppm).

Carbon	Isolated Hydroxychalcone (ppm)	Synthesized Hydroxychalcone (ppm)	Carbon	Isolated Hydroxychalcone (ppm)	Synthesized Hydroxychalcone (ppm)
2	4.55 (d,7.6)	-	4”	2.63(dd, 16.1, 8.0)<br>2.92(dd, 16.1, 5.3)	2.53
3	3.89(m)	3.6(m)	6”	6.03(s)	6(s)
4	2.58(dd,16.1, 8.2)<br>2.89(dd 16.1, 5.3)	2.50 (dd)	B”-2	6.88(d, 1.8)	6.8
6	5.99(s)	5.12(s)	B”-5	6.69(d, 8.1)	6.71
B-2	6.78(d, 1.9)	6.5	B”-6	6.73(dd,8.1, 1.8)	6.73
B-5	6.59(d, 8.2)	6.59(d, 8.2)	1’”		
2’ , 6’	7.29(2H, m)	7.23	2’” , 6’”	7.31(2H, m)	7.28
3’ , 5’	7.24(2H, br t, 7.5)	7.28	3’” , 5’”	7.37(2H, br 1t, 7.3)	7.35
7’	6.12(dd, 15.9,1.3)	6	4’”	7.30 (m)	7.3
8’	6.28(dd, 15.9, 1.3)	6.59	7’”	4.88 (d,11.0)	4.8
9’	3.27 (ddd, 5.9, 1.5, 1.3)	3.19	8’”	2.38 (ddd, 11.0, 1.5, 2.3)	2.39
3-OH	3.97(d, 4.6)	3.89	9’”	5.47(d,2.3)	5.5
5-OH	8.38 (br s)	8.59	3”-OH	4.13 (d,4.6)	4.05
2”	4.81(d,7.3)	4.16	5”-OH	8.65 (br s)	8.09
3”	4.02(m)	4.02			

In the ^{1}H NMR spectrum, the signals arising from catechin B- and C-rings were similar to those of Catechin; however the absence of signals due to A- ring indicated that cinnamaldehyde is attached to catechin at C- 6 and C- 8 positions.

The presence of 2 monosubstituted benzene rings, two oxygenated methines (H-7' and H-7"), and 2 sets of mutually coupled cis-olefininc protons (H-8', H-9', H-8" and H-9") was revealed.

The oxygenated methine protons H-7' and H-7" correlated with H-8' and H-8", respectively, as well as the phenyl proton signals (H-7' with H-2' and H-6' and H-7" with H-2" and H-6").

b) ^{13}C NMR spectrum of isolated and standard Hydroxychalcone

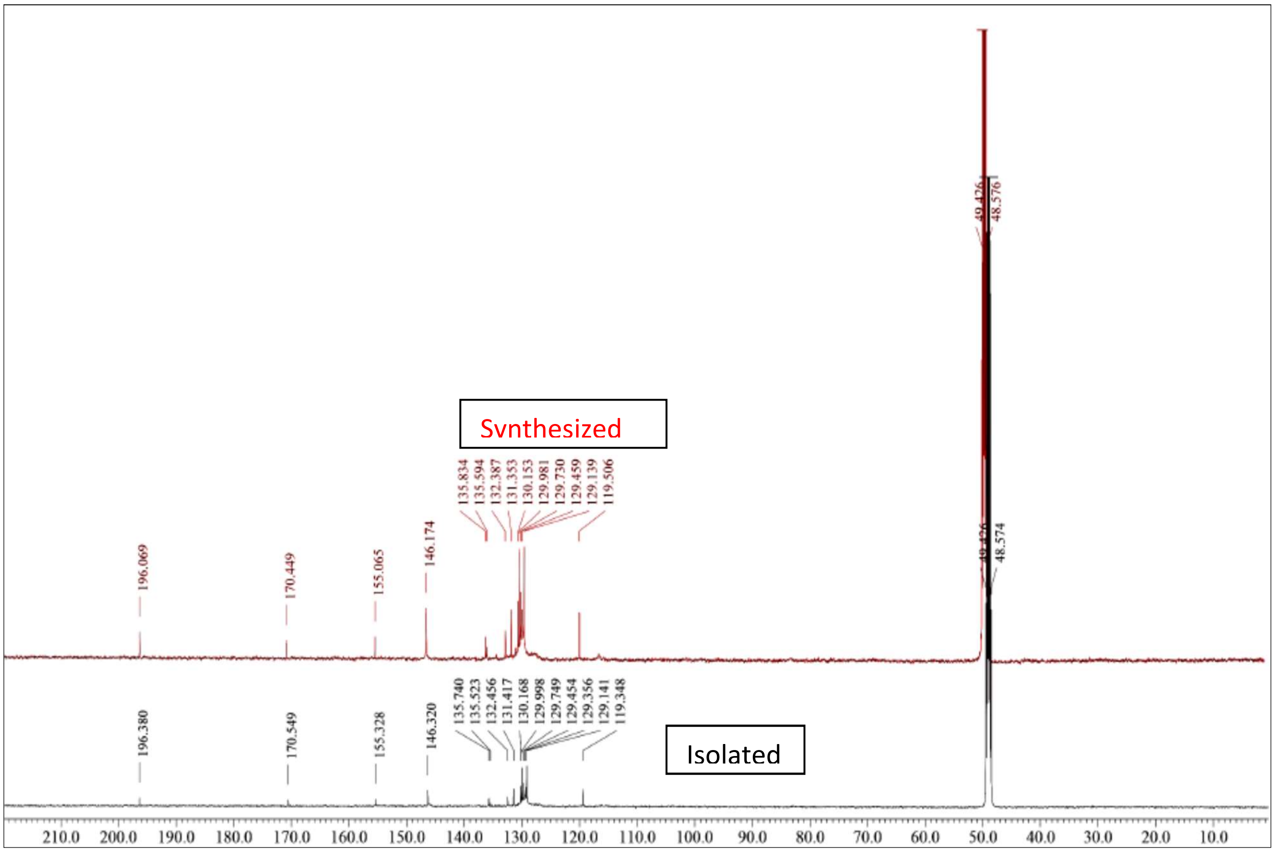

Fig.11 ^{13}C NMR spectrum of isolated and standard Hydroxychalcone

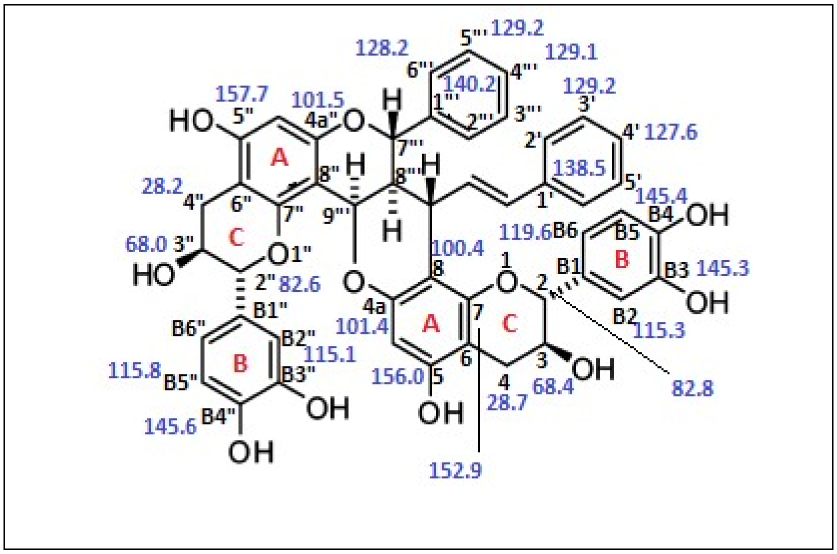

Fig 12 Structure of isolated Hydroxychalcone with the chemical shift values

Table 5 Correlation of ^{13}C NMR values of isolated Hydroxychalcone with the synthesized Hydroxychalcone (ppm).

Carbon	Isolated Hydroxychalcone (ppm)	Synthesized Hydroxychalcone (ppm)	Carbon	Isolated Hydroxychalcone (ppm)	Synthesized Hydroxychalcone (ppm)
**2**	82.8	81.24	**8'**	133.8	132.38
**5**	156.0	155.06	**9'**	33.8	33.8
**6**	95.8	100.2	**5"**	157.7	155.06
**7**	152.9	155.06	**7"**	155.2	155
**8**	100.4	120	**B"2**	115.1	119.506
**8a**	153.9	155.06	**B"3**	145.5	146.147
**B2**	115.3	119.02	**B"4**	145.6	145.2
**B3**	145.3	146.17	**B"5**	115.8	119.50
**B4**	145.4	146.18	**B"6**	119.3	119.3

**B5**	115.6	119.1	**1’’’**	140.2	138.83
**B6**	119.36	119.50	**2’’’, 6’’’**	128.2	129.12
**1’**	138.5	135.83	**3’’’, 5’’’**	129.2	129.4
**2”,6’**	127.0	129.13	**4’’’**	129.1	129.1
**3”,5’**	129.2	128.6	**7’’’**	77.4	80.49
**4’**	127.6	129.13	**8’’’**	41.4	45.6
**7’**	131.1	131.35	**9’’’**	62.7	49.42

In the 13C NMR spectrum, H-7’ and H-7” showed 1H-13C long range coupling with the C-5 and C-7 of the catechin A-ring in the spectrum. C-7’ and C-7” were attached to the catechin C-5 and C-7 through the ether linkages. The signals at h=9’ and H-9” were correlated with the catechin A-ring carbon signals (C-5, C-6, C-7, C-8 and C-8a).

These spectroscopic data indicated that Hydroxychalcone formed was a condensation product produced by coupling between A-ring C-6 and C-8 of the catechin and 2 molecules of the cinnamaldehyde.

1H and 13C NMR analysis has unequivocally established the structure of Hydroxychalcone by comparing the peaks obtained using NMR spectral data base. Catechin and the cinnamaldehyde are the flavonoids which help in scavenging the free radicals, which are the main causative factor for the degenerative disease like type 2 diabetes mellitus.

1.4 HRLCMS analysis of isolated compounds

High resolution liquid chromatographic mass spectrometry is an analytic technique that finds the relative masses of molecular ions. It is a powerful method as it provides a great deal of information like determination of molecular mass of compound, finding out the structure of the unknown substances.

1) HRLCMS of Diosgenin.

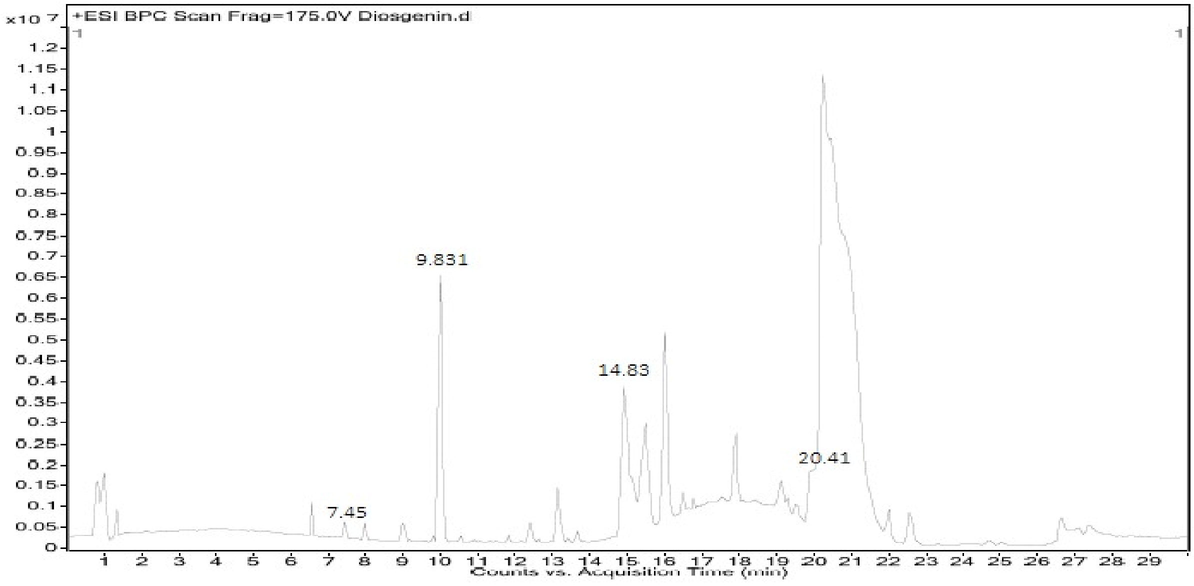

Fig 13 Mass spectrum of the diosgenin showing fragmentation peaks

The fragmentation pattern showed molecular ion peak of the compound at m/z value of 415.32 and base peak at 397.311. The mass spectrum showed peaks at m/z values of 415.32(M+), 397.11, 299.29, and 271.20.

Compound Label	Name	*m/z*	RT	Algorithm	Mass
Cpd 45: Diosgenin	Diosgenin	415.3204	14.834	Find by Molecular Feature	415.3204

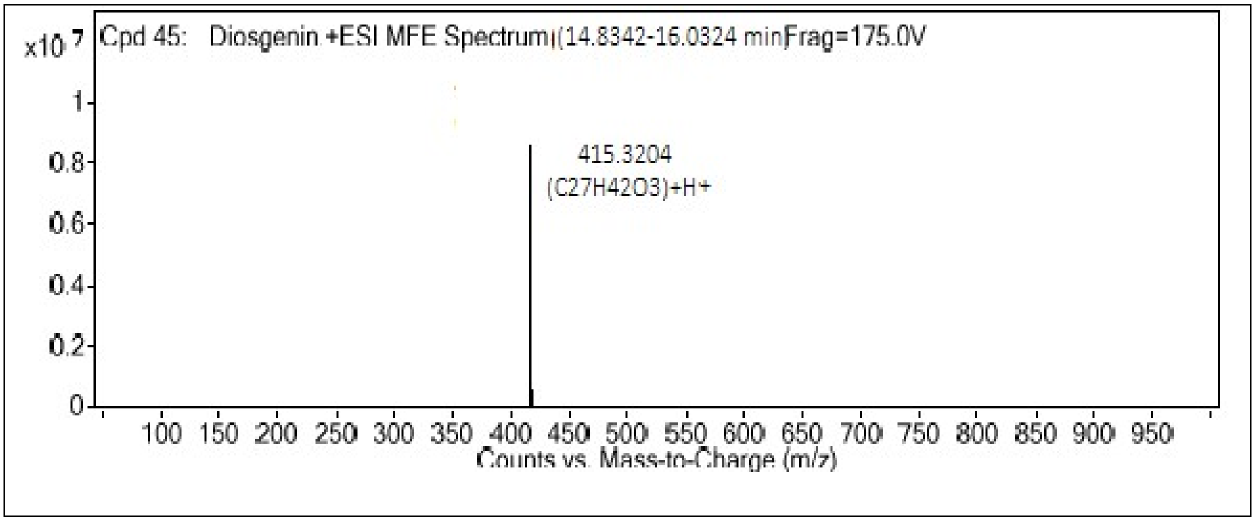

Fig 14 Molecular ion peak of Diosgenin

The molecular ion of m/z value of 415.32 undergoes loss of 57.17 mass units to give the fragment of m/z value 397.311. This fragment of 57.11 mass units can be considered as C_3H_4O. The peak for the m/z 57.17 is not recorded as it may be further fragmented before it reaches the detector.

Compound Label	Name	m/z	RT	Algorithm	Mass
Cpd 6:Bufanolide	**Bufanolide**	397.1184	9.831	Find by Molecular Feature	358.1552

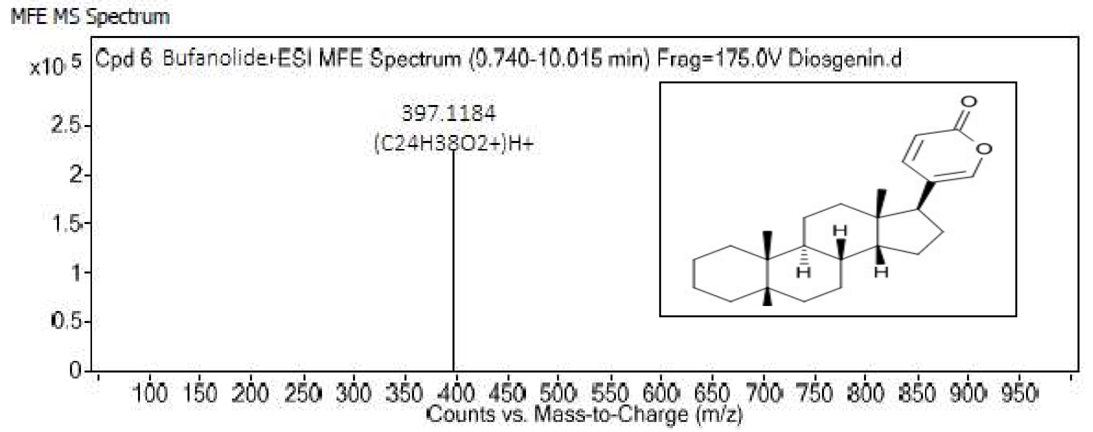

Fig 15 Mass fragmentation of Diosgenin.

The fragment of m/z 397.311 units, after further fragmentation gave another peak at m/z 299.29 showing loss of 98.021 mass units i.e., C_6H_{26} group.

Compound Label	Name	m/z	RT	Algorithm	Mass
C6H26	C6H26	299.0855	7.45	Find by Molecular Feature	98.021

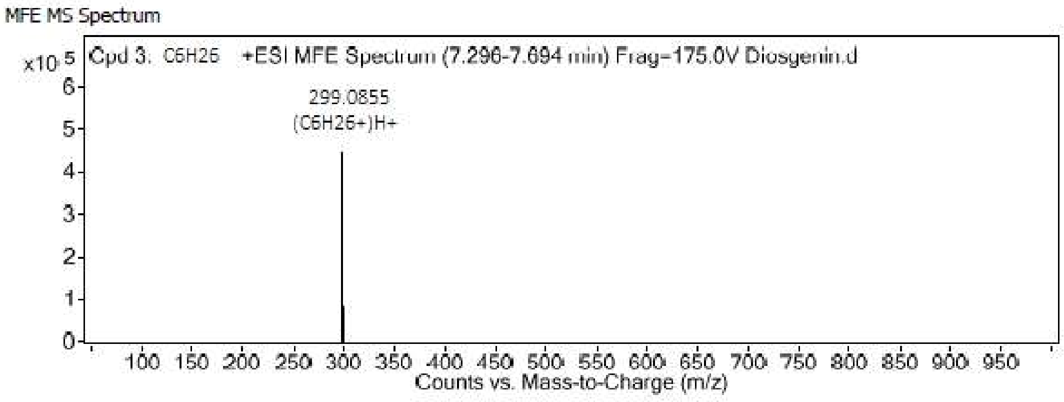

Fig 16 Mass fragmentation of Diosgenin

The base peak of m/z 397.311 units, after further fragmentation gave another peak at m/z 271.20 showing loss of 126.11 mass units i.e., C_8H_{30}.

Compound Label	Name	m/z	RT	Algorithm	Mass
Cpd C8H30	C8H30	271.182	20.415	Find by Molecular Feature	126.11

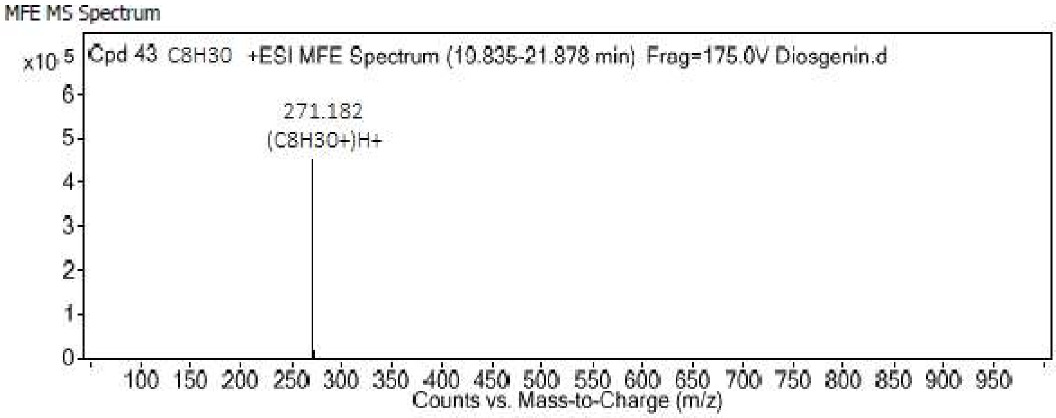

Fig 17 Mass fragmentation of Diosgenin

Both the standard as well as isolated Diosgenin were giving the same pattern of the mass fragmentation indicating the structure of the active marker compound Diosgenin.

2) HRLCMS of Hydroxychalcone.

The condensation reaction of catechin and cinnamaldehyde occurred when the mixture was heated at 100°C for 50mins. In the experiment separation of the product from sephadex LH-20 column chromatography yielded a Hydroxychalcone. Mass spectrum showed the molecular ion peak at m/z 809, which suggested that Hydroxychalcone was composed of two molecules of catechin and two of cinnamaldehyde, the molecular formula was deduced to $C_{48}H_{40}O_{12}$.

Compound Label	Name	m/z	RT	Algorithm	Mass
Cpd : Hydroxychalcone	**Hydroxychalcone**	809	21.8	Auto MS/MS	809.59

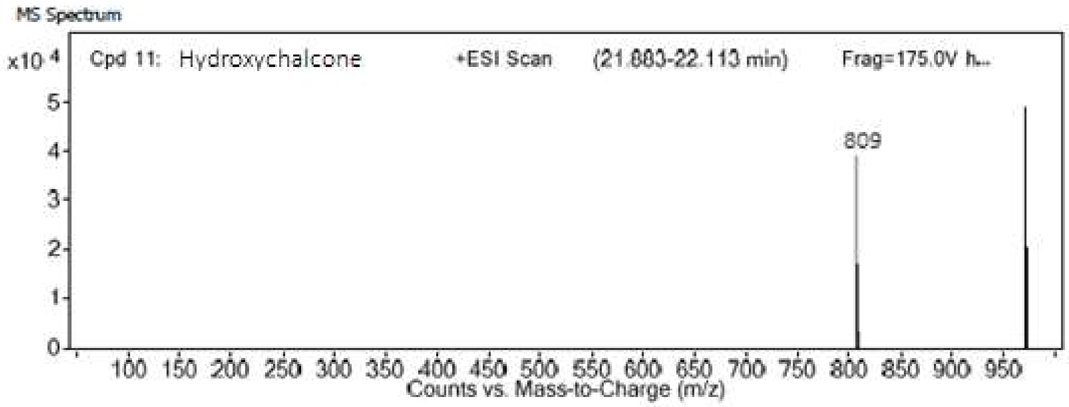

Fig 18 Mass fragmentation of isolated Hydroxychalcone

The presence of ion at m/z 287 appears to be indicative of the doubly linked Procyanidins oligomers of the Catechin/Epicatechin.

Compound Label	Name	m/z	RT	Algorithm	Mass
Cpd 42: Phthalic acid Mono-2-ethylhexyl Ester	**Phthalic acid Mono-2-ethylhexyl Ester**	287	16.898	Auto MS/MS	301.1423

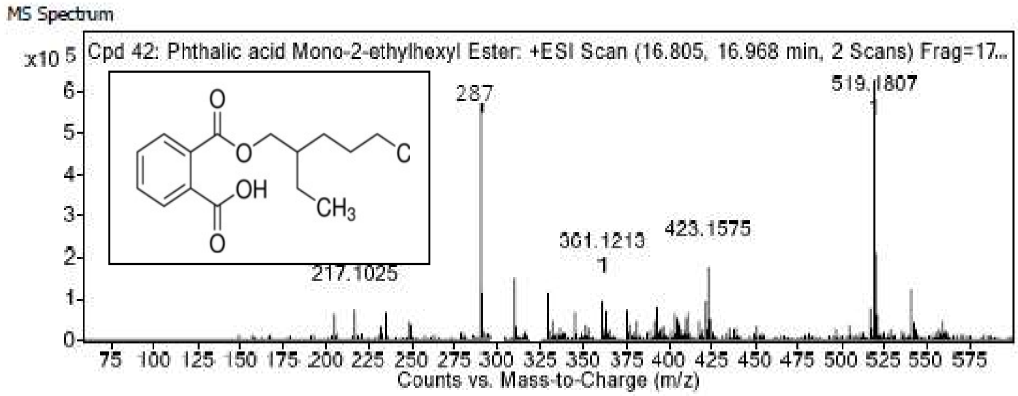

Fig 19 Mass fragmentation of isolated Hydroxychalcone

The molecular ion peak at m/z 435.2 corresponds to Ellagic acid 3-O- pentoside.

Compound Label	Name	m/z	RT	Algorithm	Mass
Cpd: Ellagic acid 3-o-pentoside	Ellagic acid 3-O-pentosdie	435.2	19.124	Find by Molecular Feature	440.6

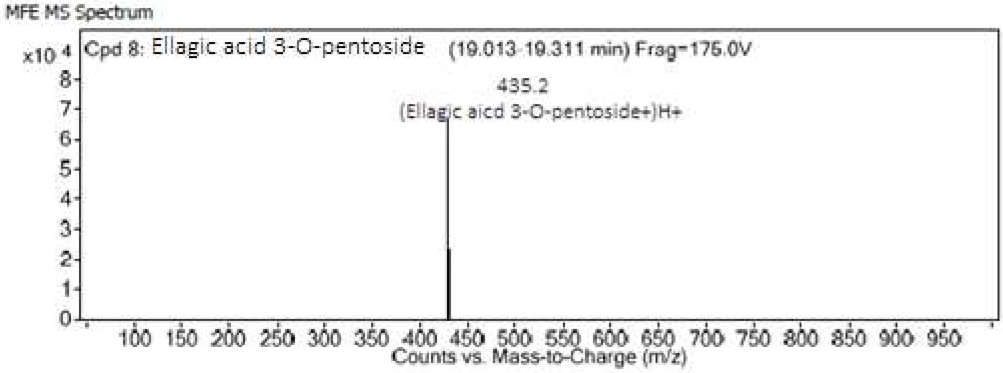

Fig 20 Mass fragmentation of isolated Hydroxychalcone

Upon further fragmentation of polyphenols the intense peak obtained at 437.2 that correspond to the Afzelechin 3-O-glucopyranoside.

Compound Label	Name	m/z	RT	Algorithm	Mass
Cpd: Afzelechin 3-O-glucopyranosisde	Afzelechin 3-O-glucopyranoside	437.2	19.124	Find by Molecular Feature	437

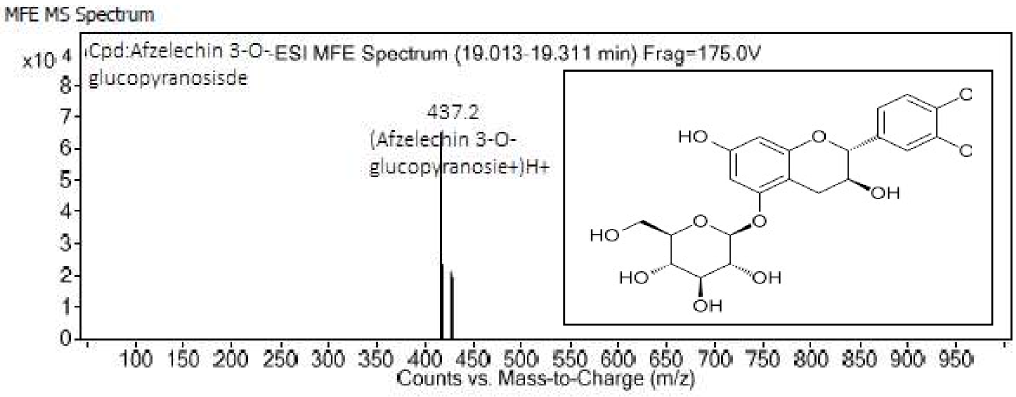

Fig 21 Mass fragmentation of isolated Hydroxychalcone

The molecular ion peaks obtained from the isolated Hydroxychalcone were found to be identical with that of the synthesized Hydroxychalcone [80, 81].

1.5 Elemental analysis of the isolated biomarkers.

The spectral studies of the marker compounds were also supported by the elemental analysis.

1) CHNO analysis of the Diosgenin.

The molecular formula for Diosgenin is $C_{27}H_{42}O_3$. From the formula, the calculated Elemental Quantities was found to be Carbon 78.21%, Hydrogen 10.21%, and Oxygen 11.58%.

According to the Elemental analysis performed, the results of the elemental quantities were as follows;

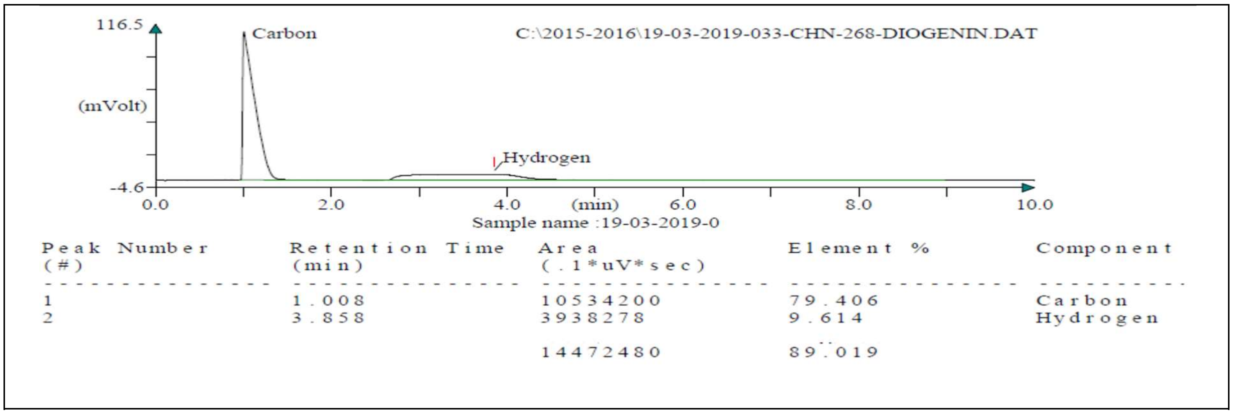

Fig 22 Carbon, Hydrogen analysis of Diosgenin

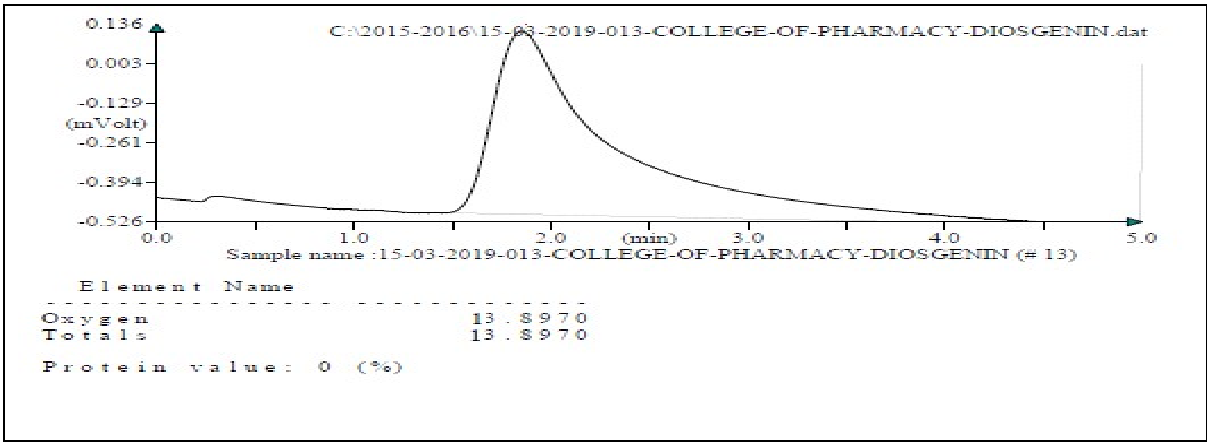

Fig 23 Oxygen analysis of Diosgenin

Both the calculated and obtained values for the elemental analysis (CHNO analysis) of the Diosgenin were found to be similar.

2) CHNO analysis of the Hydroxychalcone.

The molecular formula for Charantin is $C_{48}H_{41}O_{12}$. From the formula, the calculated Elemental Quantities was found to be Carbon 71.19%, Hydrogen 5.06%, and Oxygen 23.73%.

According to the Elemental analysis performed, the results of the elemental quantities were as follows;

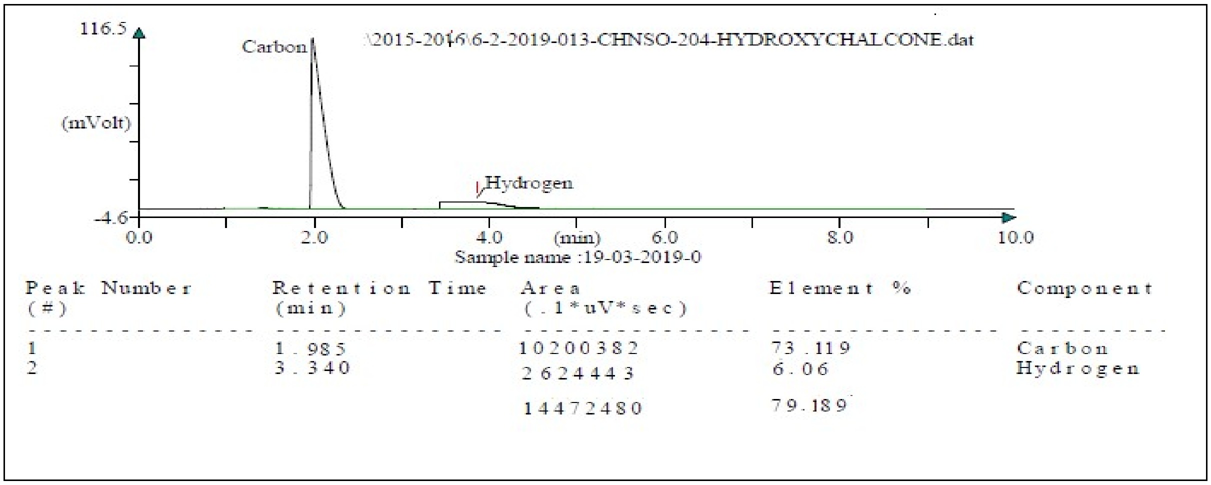

Fig 24 Carbon and Hydrogen analysis of Hydroxychalcone

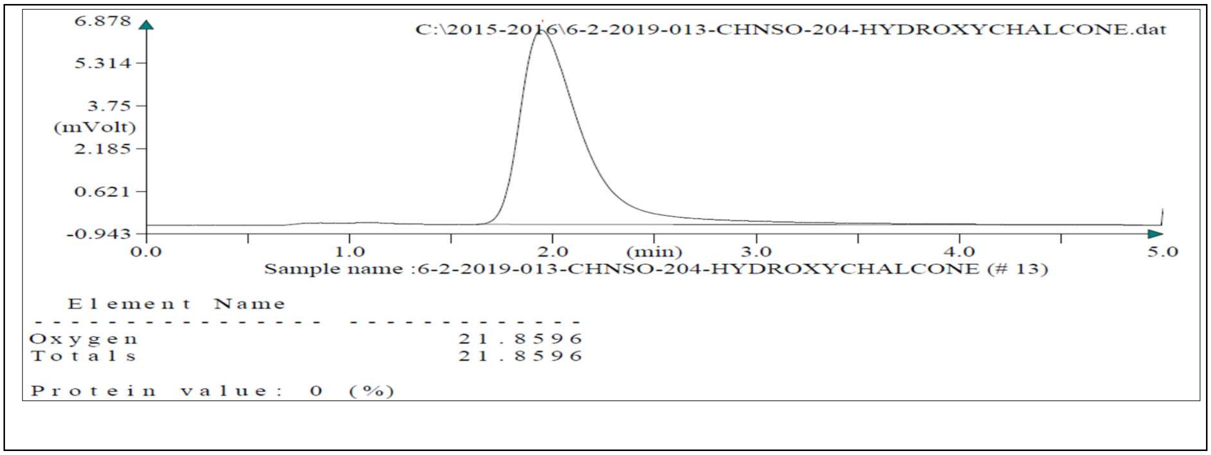

Fig 25 Oxygen analysis of Hydroxychalcone

Both the calculated and obtained values for the elemental analysis (CHNO analysis) of the Hydroxychalcone were found to be similar.

To confirm the identity of the isolated markers viz. Diosgenin and Hydroxychalcone the elemental analysis was carried out along with the spectral studies. The results are presented in the Table 4.11

Table 6 Elemental analysis of the marker compounds.

Name of the Marker compound	% Carbon		% Hydrogen		% Oxygen	
	Theoretical	Result obtained	Theoretical	Result obtained	Theoretical	Result obtained
Diosgenin	78.21	79.406	10.21	9.614	11.58	13.89
Hydroxychalcone	71.19	73.119	5.06	6.06	23.73	21.85

The results of elemental analysis were found to be coinciding with the theoretical values and the spectral data are also matching with the structural features, hence it can be concluded that the marker compounds confirm to their identity and can be utilized for further studies.

2 Pre-Clinical Evaluation of Herbal Formulations for Hypoglycemic Activity

The preclinical evaluation of the formulations for their hypoglycemic activity was carried out in streptozotocin induced diabetes model in Wistar rats. Hyperglycemia was induced in the rats by administering single dose of Streptozotocin (dose 60mg/kg b.w, i.p). The induction of hyperglycemia was confirmed through determination of the fasting blood glucose on day 3rd. The animals indicating blood glucose values above 250mg/dL were selected for the study. One set of seven rats were kept as control group.

Administration of streptozotocin leads about threefold increase in the fasting blood glucose values as compared to the healthy animals. This significant increase ($P<0.05$) in the plasma glucose value as compared to the control group indicates simulation of diabetes condition in the animals on third day. The hyperglycemic conditions were observed to be sustained till the end of study.

The rats were then treated with the different formulations, and on third day of treatment the blood glucose value indicated that treatment with formulation containing piperine could significantly ($P<0.01$) reduce blood glucose value by 36% as compared to the formulation without piperine (2.51%). The marketed herbal formulation showed significant reduction in the blood glucose level by 28%.

On the 10th and 15th day of the blood glucose analysis, the blood glucose value for the treatment with the formulation containing piperine there was significant reduction in the blood glucose values by 46% and 61% respectively as compared to that of the treatment with the herbal formulation without piperine which was 28.12% to 41% respectively. The blood glucose reduction for the treatment with marketed herbal formulation was found to be 48.28% and 56.97% on 10th and 15th day respectively of the treatment.On the 17th day of the treatment the blood glucose value indicated that the treatment containing piperine had significantly ($P<0.01$) reduced the blood glucose value by 75% as compared to the formulation without piperine (57.47%). The blood glucose reduction due to the marketed herbal formulation was found to be 84% on the 17th day. The pharmacodynamic effects of the formulation containing piperine revealed that there was significant reduction in the glucose levels as compared to the animals treated with formulation lacking piperine. There was significant increase ($P<0.05$) in the blood glucose level of all STZ treated diabetic group upto 300.52 mg/dl on 3rd day of STZ administration. This increased blood glucose level confirmed the diabetes. Treatment with polyherbal formulation with and without piperine was significantly

($p<0.05$, $p<0.01$) decrease blood glucose levels in diabetic rats compared to the STZ induced diabetic group of rats on 15th and 17th day of protocol.

Table 7 Effect of polyherbal formulations on the blood glucose level in Streptozotocin induced diabetic rats.

Sr. No	Group	Fasting Blood Glucose (mg/dl) (% reduction in blood glucose value)			
		**3rd day**	**10th day**	**15th day**	**17th day**
**1**	**Normal control (No treatment)**	92.02±1.06	92.28±1.30	93.51±0.78	92.59±1.26
**2**	**Streptozotocin induced diabetic control (60mg/kg b.w. I.p.)**	292.31±2.40**	287.89±2.74**	291±2.07**	290.18±1.16**
**3**	**Streptozotocin induced diabetic + herbal formulation (which contains piperine) 76.5mg/kg body weight p.o**	220.98±1.02** **(36%)**	199.59±2.09@@ **(46%)**	170.84±2.90@@ **(61%)**	141.40±1.8@@ **(75%)**
**4**	**Streptozotocin induced diabetic + herbal formulation (without piperine) 75mg/kg body weight p.o**	287.30±6.28** **(2.51%)**	232.821±2.25@ **(28.16%)**	210.12±1.46@ **(41%)**	177.24±3.03@ **(57.47%)**
**5**	**Streptozotocin induced diabetic + Marketed herbal formulation(Quanto Diab forte) 75mg/kg body weight p.o**	235.68±1.24** **(28.28%)**	205.23±3.98@@ **(42.258%)**	178.53±1.32@@ **(56.97%)**	123.04±2.12@@ **(84.7%)**

The results were expressed as Mean ±SD (n=7)

**p<0.01 when compared to control group of rats, @p<0.051, @@p<0.01 when compared to STZ induced diabetic group.

The study is an indirect indication of the bioavailability of plant constituents reflected through the efficiency and the study reveals that the incorporation of piperine in the formulation leads to significant ($p<0.01$) reduction in glucose levels as compared to the formulation without piperine. Also the Marketed formulation leads to the significant reduction in blood glucose values ($p<0.01$) when compared to the STZ induced diabetic group. The significant reduction in blood glucose values (84%) due to the marketed formulation as compared to the herbal formulation containing piperine (75%) on the 17th day of treatment was may be

due to the fortification of the marketed formulation with 20% amino acids and 40% saponins from the fenugreek.

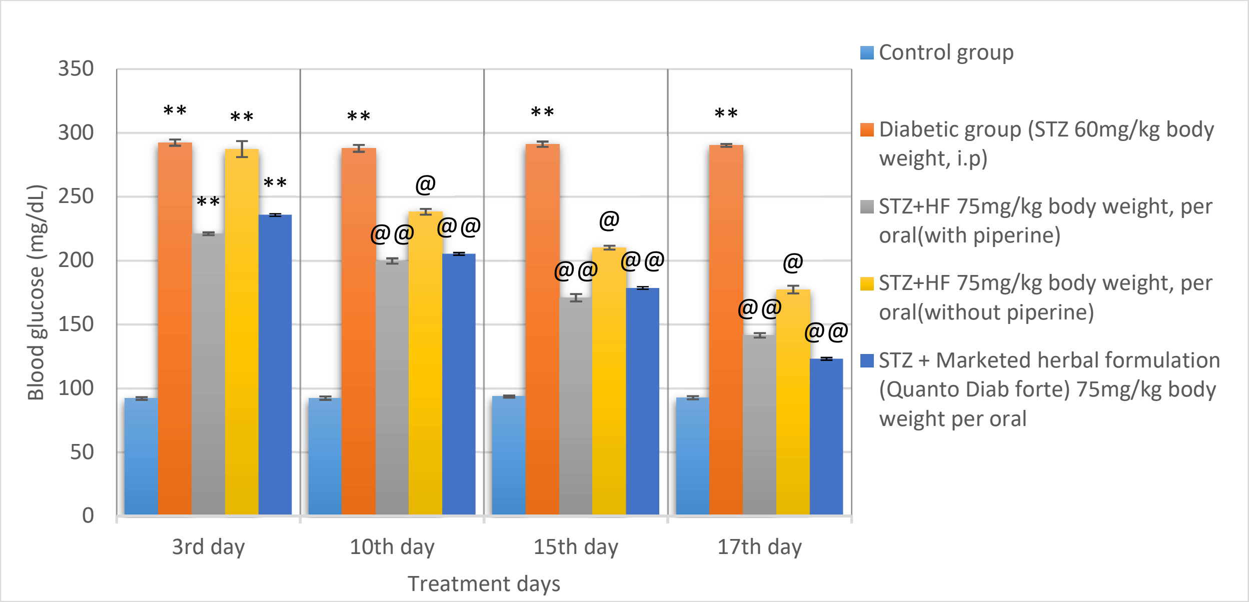

Fig 26 Effect of herbal formulation with and without piperine on blood glucose level in STZ induced diabetic rats. (Results are presented as mean ± s.d, number of rats in each group =7)

** $p<0.01$ when compared to control group of rats, @ $p<0.051$, @@ $p<0.01$ when compared to STZ induced diabetic group.

Table 7 Effect of herbal formulation on the serum creatinine, serum albumin and triglyceride level in Streptozotocin induced diabetic rats.

Sr no	Group	3rd day			10th day			17th day		
		Creatinine	Albumin	Triglycerides	Creatinine	Albumin	Triglycerides	Creatinine	Albumin	Triglycerides
1	Normal control (No treatment)	0.40±0.016	2.92±0.334	62.77±1.84	0.37±0.10	2.96±0.58	61.62±0.83	0.38±0.038	2.94±0.96	61.64±0.97
2	Streptozotocin induced diabetic control (60mg/kg b.w.i.p.)	0.72±0.27*	3.93±0.60*	116.21±3.75*	0.78±0.17**	4.22±1.28*	120.7±1.58**	0.71±0.08*	4.07±1.09**	111.74±2.20*
3	Streptozotocin induced diabetic + herbal formulation (whichcontains piperine) 75mg/kg body weight p.o	0.65±0.07 **(96.4%)**	3.21±0.50@@ **(71.29%)**	93.75±3.20 **(43.43%)**	0.49±0.13@@ **(70%)**	3.21±0.76@ **(80.16%)**	81.58±1.93@@ **(66.22%)**	0.39±0.13@@ **(96.97%)**	3.16±0.44@@ **(80.53%)**	67.32±2.98@@ **(88.66%)**
4	Streptozotocin induced diabetic +herbal formulation (without piperine) 75mg/kg body weight p.o	0.70±0.06 **(2.95%)**	3.52±0.67 **(40.59%)**	99.94±0.94 **(30.45%)**	0.59±0.14@ **(9.59%)**	3.52±0.96@ **(55.56%)**	85.21±1.22@ **(60.07%)**	0.46±0.023@@ **(75.76%)**	3.49±0.42@ **(51.33%)**	72.73±2.03@@ **(77.86%)**
5	Streptozotocin induced diabetic +Marketed herbal formulation (QuantoDiabforte) 75mg/kg body weight p.o	0.69.90±0.23@ **(7.45%)**	3.31±0.32@@ **(61.39%)**	96.32±2.06 **(37.22%)**	0.51±0.09@@ **(65.68%)**	3.35±0.58@@ **(69.05%)**	83.29±0.92@@ **(63.32%)**	0.42±0.03@@ **(87.88%)**	3.20±0.36@@ **(70.80%)**	69.23±1.20@@ **(84.85%)**

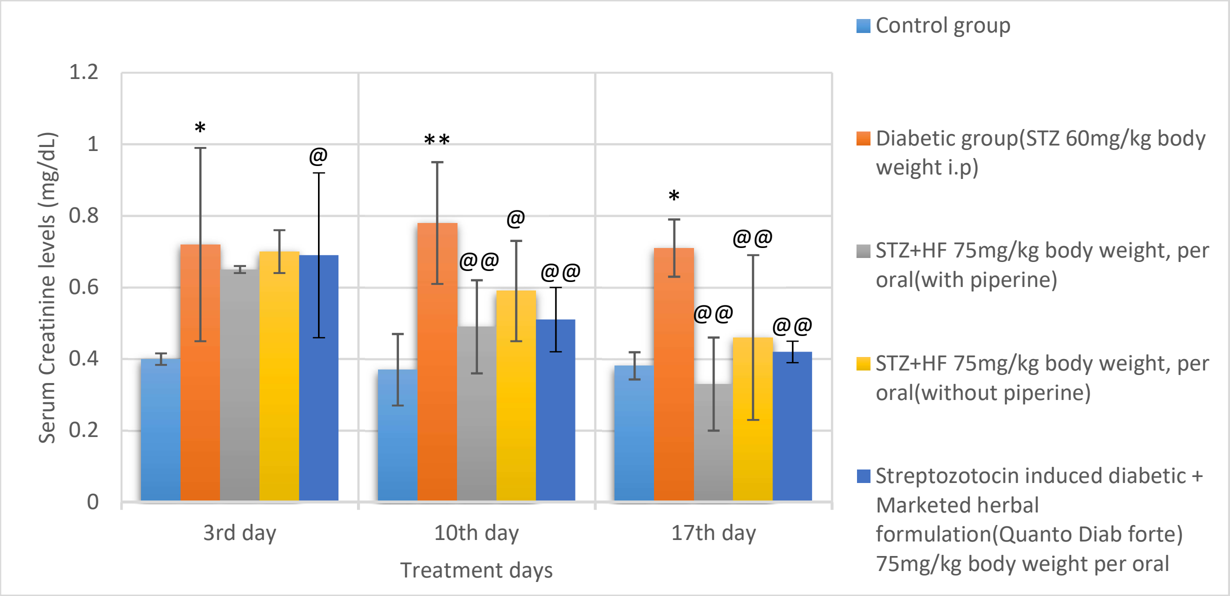

Fig 27 Effect of herbal formulation with and without piperine on serum Creatinine level in STZ induced diabetic rats. (Results are presented as mean ± s.d, number of rats in each group =7)

**P<0.01 when compared to the control group of the rats, @P<0.05, @@P<0.01 when compared to the negative control group

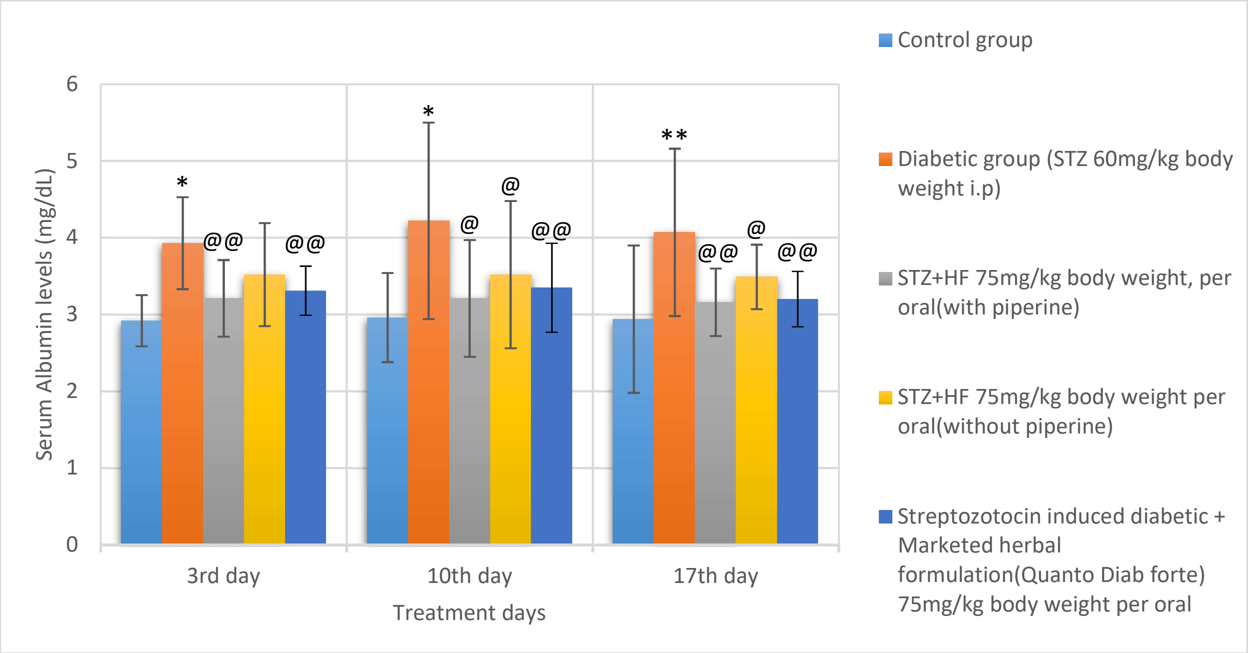

Fig 28 Effect of herbal formulation with and without piperine on serum Albumin level in STZ induced diabetic rats. (Results are presented as mean ± s.d, number of rats in each group =7) **

P<0.01 when compared to the control group of the rats, @P<0.05, @@P<0.01 when compared to the negative control group.

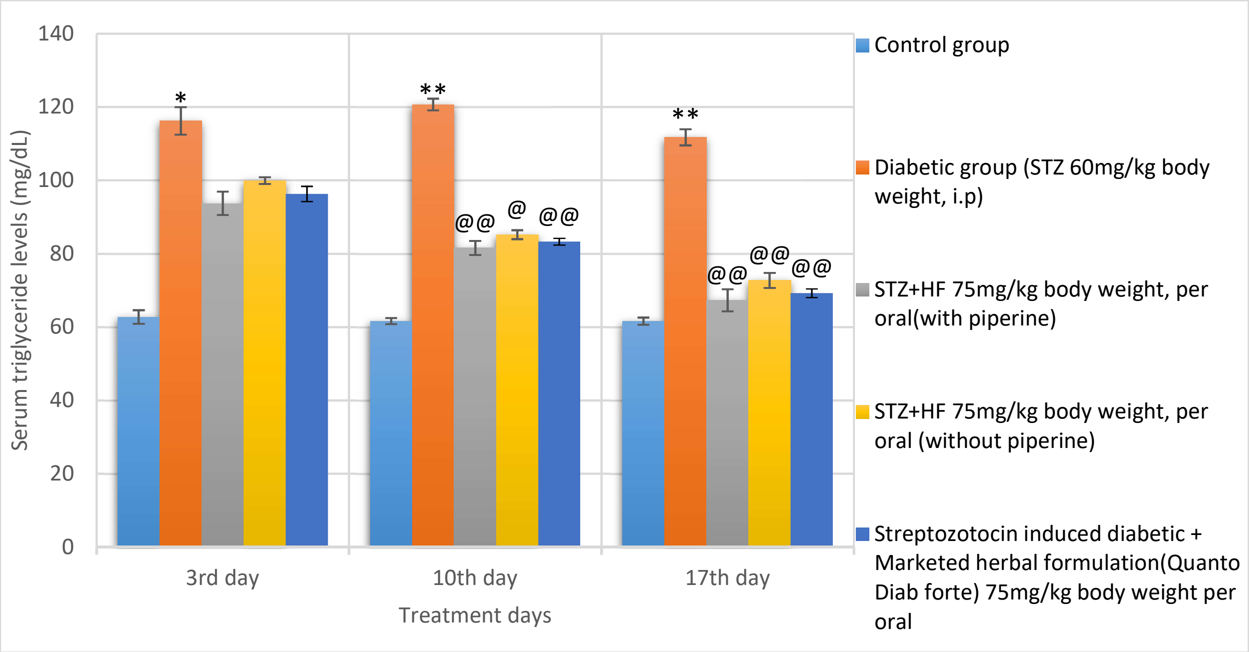

Fig 29 Effect of herbal formulation with and without piperine on serum Triglycerides level in STZ induced diabetic rats. (Results are presented as mean ± s.d, number of rats in each group =7)

**P<0.01 when compared to the control group of the rats, @P<0.05, @@P<0.01 when compared to the negative control group.

Effect of poly herbal formulations on the serum concentration of creatinine, serum albumin and triglyceride in STZ induced diabetic rats shown in Table 7 there was twofold increase in the concentrations of the creatinine, albumin and triglyceride level in the serum of STZ induced diabetic rats as compared to that of the control group of rats. However, treatment with herbal formulation with piperine significantly (p<0.05) decreases the serum concentration of creatinine (70%) as compared to that of the formulation without piperine (9.59%), also decreases the concentration of serum albumin and triglyceride (80.16%, 66.22% respectively) as compared to the formulation without piperine (55.65%, 60.07% respectively) on the 10th and 17th day of protocol.

Streptozotocin is widely used to induce the experimental diabetes in animals. It is reported that the streptozotocin enters the β-cells via glucose transporter (GLUT2) and causes the alkylation of DNA. DNA damage induces activation of poly ADP-ribosylation leads to depletion of cellular NAD+ and ATP. Enhanced ATP dephosphorylation after streptozotocin treatment supplies a substrate for xanthine oxidase

resulting in formation of superoxide radicals. As a result of the streptozotocin action, β-cells undergo the destruction by necrosis.

Diosgenin is a sapogenin obtained from *T foenum-graecum* is reported to have protective effect on pancreatic β-cells, down regulation of enzymes involved in hepatic gluconeogenesis and glucose export, upregulation of hepatic glucokinase, and increases in the amounts of hepatoprotective and antioxidant enzymes.

Charantin is a steroidal sapogenin obtained from *M charantia* is reported to have protective effect on beta cells, inhibition of glucose uptake inhibition of adipocyte differentiation, suppression of gluconeogenic enzymes.

Hydroxychalcone is a flavonoid obtained from the *C zeylanicum;* it is reported to elevate insulin sensitivity as well as to lower insulin resistance.

Piperine is an alkaloid obtained from *P nigrum* is reported to have bioenhancing activity; it causes increased gastrointestinal absorption by enhancing the secretion of bile acids which leads to the increase in solubility and absorption. Piperine by interacting with the intestinal epithelial cells stimulates gamma-glutamyl transpeptidase activity and causes as increase in amino acid uptake by epithelial cells. Also it has been reported that piperine has inherent antidiabetic activity, which may be responsible for the significant reduction in the glucose levels due to formulation containing piperine as compared to the formulation without piperine.

Other constituents present in all plants and all these drugs when combined they produce synergistic effects.

3 Relative Bioavailability.

Relative bioavailability measures the bioavailability of a formulation of certain drug when compared with another formulation of the same drug. Relative bioavailability is one of the measures used to assess bioequivalence between two drug products. Table 8 to Table 10 represents the relative bioavailability of the marker compounds, viz. Diosgenin, Charantin, and Hydroxychalcone in the formulation without piperine, formulation with piperine, marketed herbal formulation (Quanto Diab Forte Capsules), and oral pure drug administration respectively.

Table 8 Relative bioavailability of the Diosgenin, in the formulation without piperine, formulation with piperine, marketed herbal formulation, and oral pure drug administration.

**Formulations**	**AUC^{0}_{∞} (µg.h/ml)**	**Dose (mg/kg p.o)**	**Relative bioavailability**		
			**Standard**	**Sample**	**Relative bioavailability**
Formulation without piperine	1.9838	0.68	Formulation with piperine	Formulation without piperine	0.7346
Formulation with piperine	2.7006	0.68	Marketed herbal formulation	Formulation without piperine	1.3928
Marketed herbal formulation	2.1532	1.028	Marketed herbal formulation	Formulation with piperine	1.8961
Oral pure drug administration	4.4753	15	Oral pure drug administration	Formulation without piperine	9.7782
			Oral pure drug administration	Formulation with piperine	13.3113
			Oral pure drug administration	Marketed herbal formulation	8.1941

Result presented in Table 8 indicates that the relative bioavailability of the formulation without piperine when compared to that of formulation with piperine was found to be 0.73. When formulation without piperine was compared with that of the marketed herbal formulation it was found to be 1.39. At the same time, when relative bioavailability of the formulation with piperine when compared to that of the marketed herbal formulation it was found to be 1.89. This indicates relative bioavailability of the formulation enhanced due to the presence of piperine. Relative bioavailability for the formulation containing piperine when compared to the pure drug was found to be 13.31. When relative bioavailability for the marketed herbal formulation compared to that of pure oral drug it was found to be 8.19. Hence, it can be concluded that there is 13 fold increases in the bioavailability of Diosgenin due to presence of piperine.

Table 9 Relative bioavailability of the Charantin, in the formulation without piperine, formulation with piperine, marketed herbal formulation, and oral pure drug administration.

**Formulations**	**AUC^{0}_{∞} (µg.h/ml)**	**Dose (mg/kg p.o)**	**Relative bioavailability**		
			**Standard**	**Sample**	**Relative bioavailability**
Formulation without piperine	1.6009	0.179	Formulation with piperine	Formulation without piperine	0.6836

Formulation with piperine	2.3419	0.179	Marketed herbal formulation	Formulation without piperine	1.6245
Marketed herbal formulation	2.8849	0.5240	Marketed herbal formulation	Formulation with piperine	2.3764
Oral pure drug administration	12.4872	15	Oral pure drug administration	Formulation without piperine	10.7433
			Oral pure drug administration	Formulation with piperine	15.7160
			Oral pure drug administration	Marketed herbal formulation	6.6134

As per the result presented in Table 9, it was indicated that the relative bioavailability of the formulation without piperine when compared to that of the marketed herbal formulation was found to be 1.62. Relative bioavailability of formulation with piperine when compared to that of the marketed herbal formulation was found to be 2.37. At the same time, when relative bioavailability of formulation with piperine was compared that of pure drug was found to be 15.71. Marketed herbal formulation when compared with that of pure drug was found to be 6.61. Relative bioavailability of the formulation has been increased due to piperine.

Table 10 Relative bioavailability of the Hydroxychalcone, in the formulation without piperine, formulation with piperine, marketed herbal formulation, and oral pure drug administration.

**Formulations**	**AUC^{0}_{∞} (µg.h/ml)**	**Dose (mg/kg p.o)**	**Relative bioavailability**		
			**Standard**	**Sample**	**Relative bioavailability**
Formulation without piperine	1.4294	0.0364	Formulation with piperine	Formulation without piperine	0.8523
Formulation with piperine	1.6772	0.0364	Marketed herbal formulation	Formulation without piperine	1.4180
Marketed herbal formulation	2.2764	0.0822	Marketed herbal formulation	Formulation with piperine	1.6638
Oral pure drug administration	72.994	15	Oral pure drug administration	Formulation without piperine	8.0701
			Oral pure drug administration	Formulation with piperine	9.4692
			Oral pure drug administration	Marketed herbal formulation	5.6912

As per the result presented in table 10, the relative bioavailability of formulation containing piperine when compared with that of marketed herbal formulation was found to be 1.66. When relative bioavailability of herbal formulation without piperine was compared with that of marketed herbal formulation was found to be 1.41. It was observed that there was increase in relative bioavaiblity of the Formulation due to presence of the piperine.

Results of the relative bioavaiblity as presented in Table 8 to 10 clearly indicate that there was increase in the bioavaiblity of the formulation due to incorporation of the piperine. Piperine was known for the enhancement of bioavaiblity of the drug components from the ancient times. Also piperine itself has inherent antidiabetic activity, which might be responsible for the significant reduction in blood glucose level and increase in the plasma concentration of marker compounds. When relative bioavailability was study it was found that there was 13 to 15-fold increase in the bioavaiblity of the marker compounds. Marketed formulation had significantly more bioavaiblity value; which may be because of the more concentration of the marker compounds present in the marketed formulation, also marketed formulation have been fortified with that of 20% of amino acid (4-Hydroxyisoleucine) and 40% of the saponin. 4-Hydroxyisoleucine was reported increase the insulin secretion thereby producing the anti-diabetic effect.
